# Reduced DDB2 Expression Is Associated with Temozolomide Resistance in Glioblastoma Cells

**DOI:** 10.3390/ijms27208941

**Published:** 2026-10-09

**Authors:** Inés Grávalos-Cano, Teresa Morales-Ruiz, Rafael R. Ariza, Teresa Roldán-Arjona, María Isabel Martínez-Macías

**Affiliations:** 1 Department of Genetics, University of Córdoba, 14014 Córdoba, Spain; 2 Maimónides Biomedical Research Institute of Córdoba (IMIBIC), 14004 Córdoba, Spain

**Keywords:** glioblastoma, DNA repair, DNA damage, temozolomide, DDB2

## Abstract

Glioblastoma (GBM) remains one of the most lethal primary brain tumours, and resistance to temozolomide (TMZ), the standard chemotherapeutic agent for GBM, represents a major obstacle to effective treatment. Although O^6^-methylguanine-DNA methyltransferase (MGMT) is a well-established determinant of TMZ response, MGMT status alone does not fully explain the heterogeneity of treatment outcomes, suggesting the involvement of additional DNA damage response mechanisms. Here, we investigated the contribution of different DNA repair pathways to TMZ sensitivity in a panel of GBM cell lines displaying distinct TMZ response profiles. Expression analysis of 84 DNA repair genes identified damage-specific DNA binding protein 2 (*DDB2*) as the only one consistently associated with TMZ sensitivity. We found that *DDB2* expression was significantly reduced in TMZ-resistant cell lines and selectively induced following TMZ exposure in TMZ-sensitive cells. Proteomic profiling of representative sensitive and resistant cell models further revealed distinct responses to TMZ and identified enrichment of p53-associated DNA damage response pathways in sensitive cells, including altered expression of DDB2 and other canonical p53-regulated proteins. Importantly, siRNA-mediated depletion of DDB2 in the TMZ-sensitive A172 GBM cell line significantly reduced TMZ sensitivity. These findings provide functional evidence that DDB2 contributes to the cellular response to TMZ and extend the established roles of DDB2 in DNA repair and cancer biology to TMZ responsiveness in GBM.

## 1. Introduction

GBM is the most aggressive primary brain malignancy in adults and remains associated with poor clinical outcomes despite multimodal treatment [1]. According to the 2021 World Health Organization classification, GBM is defined as an IDH-wildtype diffuse astrocytic glioma characterized by features including necrosis, microvascular proliferation, TERT promoter mutation, EGFR amplification, or combined chromosome 7 gain/chromosome 10 loss [2]. GBM accounts for more than half of all gliomas and is typically diagnosed in older adults [3]. The current standard of care consists of surgical resection followed by radiotherapy and chemotherapy. Although surgery remains an important component in the management of GBM, radical tumour resection is not curative due to its highly invasive nature. After primary resection, radiotherapy combined with TMZ, an alkylating agent that is the standard chemotherapeutic agent used for GBM, is the most effective adjuvant therapy for prolonging survival [4]. Despite maximal surgical resection followed by radiotherapy and TMZ-based chemotherapy, median overall survival remains approximately 15 months. This is partly due to the intrinsic or acquired resistance to TMZ that develops in a substantial proportion of patients and represents a major cause of treatment failure, tumour recurrence and poor survival [5,6].

TMZ induces several DNA lesions, primarily O^6^-methylguanine (O^6^-meG), N3-methyladenine (N3-meA) and N7-methylguanine (N7-meG), which trigger cytotoxicity through replication stress, DNA damage accumulation and cell death [7,8]. Each of these three types of DNA damage has different consequences. O^6^-meG mispairs with thymine, and has mutagenic and cytotoxic effects, whereas N3-meA blocks DNA synthesis and is cytotoxic. N7-meG, which is the predominant lesion, is innocuous by itself, but is prone to spontaneous depurination, generating abasic (apurinic/apyrimidinic, AP) sites with pronounced cytotoxic and mutagenic effects [8].

Resistance to TMZ has been in part linked to the activity of DNA repair pathways that counteract the cytotoxic DNA lesions induced by this alkylating agent [9]. Elevated expression of O^6^-methylguanine-DNA methyltransferase (MGMT), which directly reverses O^6^-meG lesions, is a well-established determinant of TMZ resistance and an important predictive biomarker in GBM [10]. Since *MGMT* promoter methylation reduces protein expression and increases sensitivity to TMZ, its methylation status is widely used as a prognostic biomarker of treatment response in GBM patients [11].

On the other hand, the Mismatch Repair (MMR) pathway is involved in the processing of O^6^-meG lesions when MGMT is absent [10]. The MutSα recognition complex [MutS Homolog 2 (MSH2) and MutS Homolog 6 (MSH6) heterodimer] binds to the O^6^-meG:T mismatch, triggering activation of the MMR pathway. During the repair process, thymine is removed but O^6^-meG remains unrepaired. A DNA polymerase subsequently reinserts a thymine opposite the lesion, resulting in repeated cycles of futile MMR. This process can lead to cell-cycle arrest and/or cell death due to the accumulation of single-stranded DNA (ssDNA) gaps and the generation of double-strand breaks (DSBs) during subsequent rounds of replication [12]. Low expression of MMR-related proteins, such as MSH2 and MSH6, has been associated with TMZ resistance as it enables tumour cells to evade DNA repair-mediated cytotoxicity and cell death [13]. Importantly, N3-meA and N7-meG are the most abundant TMZ-induced adducts and are primarily repaired through the Base Excision Repair (BER) pathway [8,14]. Elevated expression of several BER proteins, including N-methylpurine DNA glycosylase (MPG) [15,16], apurinic/apyrimidinic endonuclease 1 (APE1) [17], and poly(ADP-ribose) polymerase 1 (PARP1) [18], has been associated with TMZ resistance and poor prognosis in glioma patients. However, these repair pathways do not fully explain the heterogeneous response observed in response to TMZ, suggesting that other DNA repair mechanisms may contribute to resistance [5].

To gain further insight into the contribution of DNA repair pathways to TMZ response in GBM, we profiled the expression of a panel of DNA repair genes across six GBM cell lines displaying distinct sensitivities to TMZ. Among the genes examined, damage-specific DNA binding protein 2 (*DDB2*) was the only gene showing consistent differential expression between TMZ-sensitive and TMZ-resistant cells. DDB2 is best characterized as a lesion-recognition and chromatin-access factor in mammalian global-genome nucleotide excision repair (GG-NER) of UV-induced DNA photolesions [19]. Beyond lesion recognition, DDB2 functions as the substrate-receptor subunit of the CRL4-DDB2 E3 ubiquitin ligase complex, promoting the ubiquitination of itself, XPC, histones, and other chromatin-associated factors and thereby contributing to the regulation of chromatin accessibility, factor recruitment, and release during DNA repair [20]. Increasing evidence has also implicated DDB2 in cancer development and progression, with reported effects on cancer cell proliferation and survival, epithelial–mesenchymal transition, migration, and invasion [21]. In the present study, DDB2 emerged from an unbiased comparative analysis of DNA repair gene expression as a candidate factor associated with TMZ responsiveness in GBM. Furthermore, we show that DDB2 expression contributes to the cellular response to TMZ, extending the reported roles of DDB2 in DNA repair and cancer biology to TMZ responsiveness in GBM.

## 2. Results

### 2.1. TMZ Response Profiling Identifies Sensitive and Resistant GBM Cell Lines

**Figure 1:**
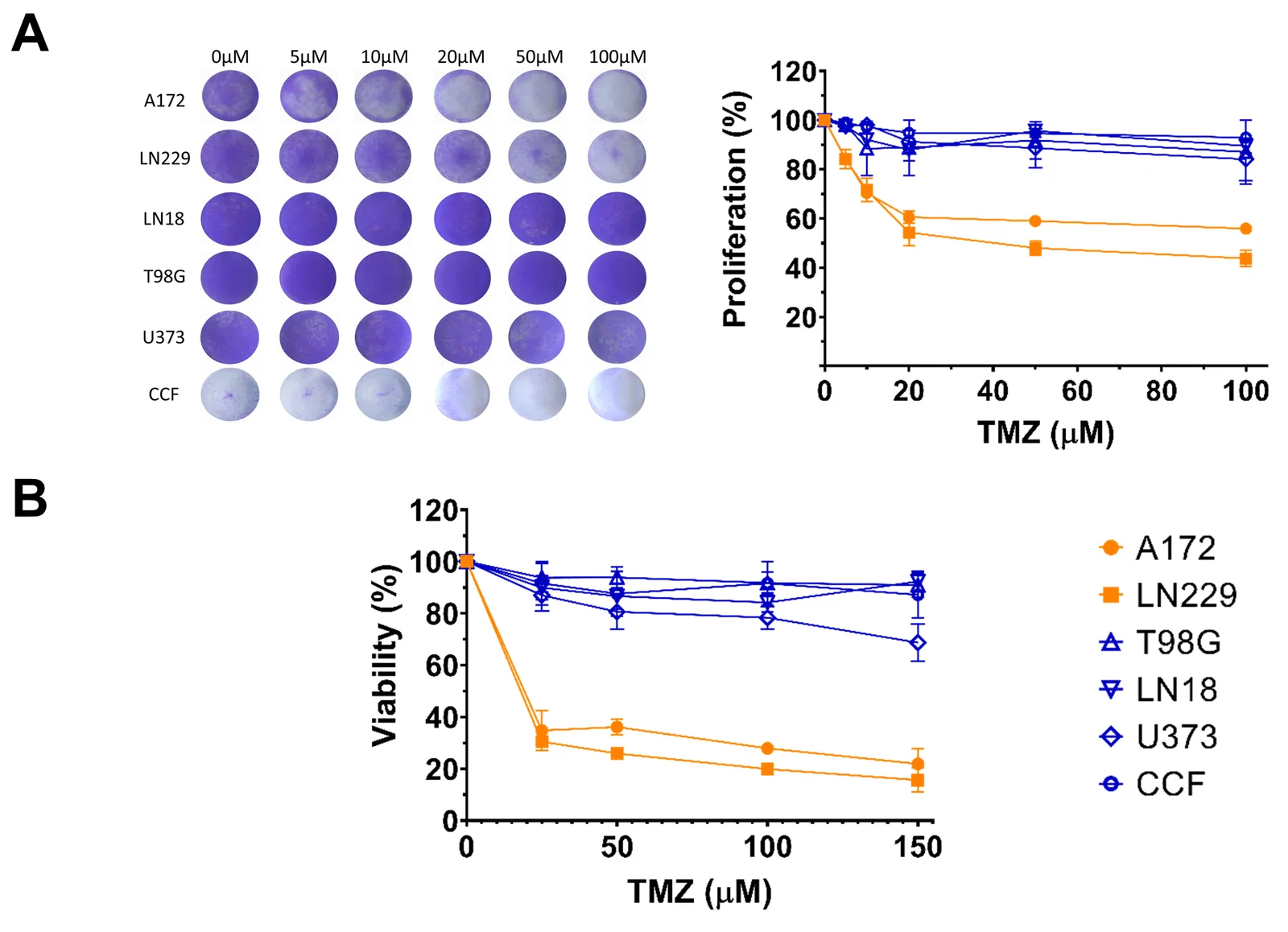
**Temozolomide sensitivity and resistance in six GBM cell lines.** (**A**) Crystal violet proliferation assay. Representative images (left) and quantification of cell proliferation (right) after treatment with increasing concentrations of TMZ. Crystal violet staining was quantitatively assessed by measuring signal intensity through image analysis. Data are mean ± SEM (*n* = 3 independent biological replicates). (**B**) WST-1 viability assay. Quantification of cell viability after treatment with increasing concentrations of TMZ. Data are mean ± SEM (*n* = 3 independent biological replicates).

To characterize the response of GBM cells to TMZ, six GBM cell lines were exposed to increasing TMZ concentrations. The panel included five commonly used GBM cell lines and CCF-STTG1 (hereafter referred to as CCF), a human astrocytoma cell line historically classified as a grade IV astrocytoma and more recently used as a model of high-grade glioma/GBM [5]. Both proliferation and viability assays revealed differences in TMZ resistance between different cell lines (Figure 1; Tables S2–S9). For both assays, two-way ANOVA revealed a significant interaction between treatment and cell line (*p* < 0.0001; Tables S2 and S5). Dunnett’s and Tukey’s post hoc analyses also revealed significant differences in TMZ resistance among cell lines, as assessed by proliferation and viability assays (Tables S3–S7).

Proliferation assays showed a pronounced reduction in cell growth in A172 and LN229 cells at doses ≥ 10 µM TMZ. In contrast, T98G, LN18, U373 and CCF cells displayed proliferation rates comparable to untreated controls across the entire TMZ dose range (Figure 1A; Tables S2–S4). Cell proliferation was quantitatively assessed by measuring the signal intensity of the crystal violet staining through image analysis; the representative images shown in Figure 1A are therefore illustrative of the staining patterns, whereas the quantitative measurements were used for the comparison of proliferation across TMZ concentrations. Although the CCF crystal violet images appeared paler than those of the other cell lines, the quantitative analysis of the staining showed the same overall pattern of TMZ response observed in the independent viability assay.

In cell viability assays (Figure 1B), TMZ treatment significantly reduced viability in A172 and LN229 cells, whereas T98G, LN18, U373 and CCF cells retained high metabolic activity even at the highest TMZ concentrations tested (Tables S5–S7). Consistent with these findings, quantitative determination of the TMZ IC_50_ values further confirmed the marked differences in TMZ sensitivity among the cell lines (Tables S8 and S9). In the proliferation assay, IC_50_ values were 61.15 μM for A172 and 38.51 μM for LN229, compared with 523.4 μM for T98G, 704.4 μM for LN18, 453.1 μM for U373 and 1060 μM for CCF. In the viability assay, the corresponding IC_50_ values were 23.17 μM and 15.24 μM for A172 and LN229, respectively, compared with 1188 μM for T98G, 792.1 μM for LN18, 301.6 μM for U373 and 832.5 μM for CCF. Thus, the two independent assays yielded concordant differences in TMZ responsiveness, with substantially lower IC_50_ values in A172 and LN229 than in the remaining cell lines. Collectively, these results support the classification of A172 and LN229 as relatively TMZ-sensitive cell models, whereas T98G, LN18, U373 and CCF were classified as relatively TMZ-resistant under the experimental conditions used in this study.

### 2.2. DNA Damage Repair (DDR) Gene Expression Profile in GBM Cell Lines

**Figure 2:**
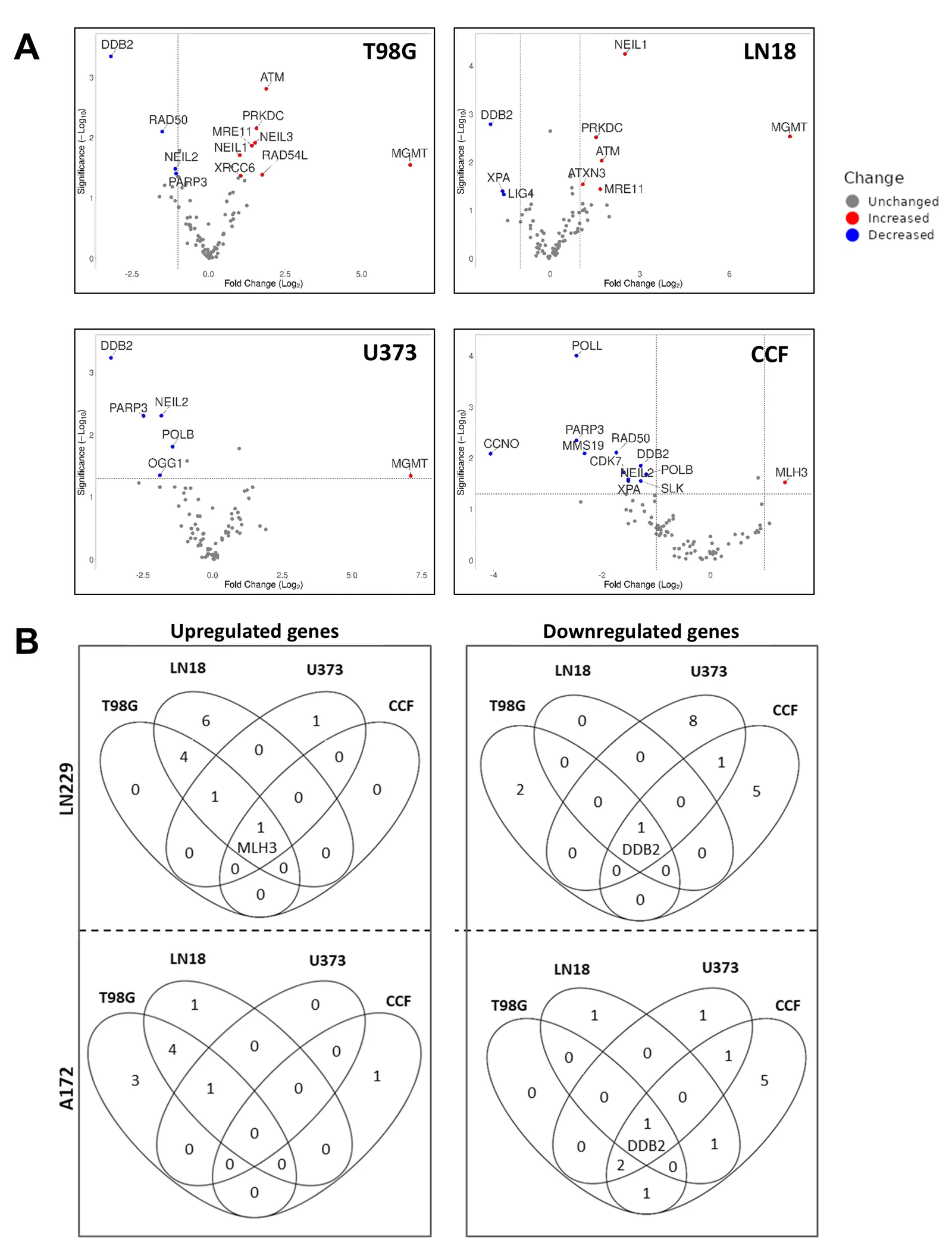
**Differential expression of DNA repair genes in TMZ-sensitive and TMZ-resistant GBM cell lines.** (**A**) Volcano plots showing differentially expressed genes (DEGs) identified by RT^2^ Profiler PCR Array analysis in TMZ-resistant GBM cell lines relative to the TMZ-sensitive A172 cell line (reference control). Genes significantly differentially expressed (*p* < 0.05) are labelled. Genes with a fold change < 2 and *p* < 0.05 are shown in blue, indicating decreased expression compared to A172, whereas genes with a fold change > 2 and *p* < 0.05 are shown in red, indicating increased expression. (**B**) Venn diagrams illustrating the overlap of DEGs between TMZ-resistant cell lines (T98G, LN18, U373 and CCF) and TMZ-sensitive cell lines LN229 (top) and A172 (bottom).

We next compared the expression of 84 DNA repair genes in TMZ-resistant and TMZ-sensitive cells using the RT^2^ Profiler PCR array. Tables S10 and S11 summarize the fold-change for those genes with significant *p*-values (based on Student’s *t*-test of the replicate 2^−ΔCT^ values for each gene), using TMZ-sensitive cell lines A172 (Table S10) or LN229 (Table S11) as controls. To facilitate the identification of differentially expressed genes (DEGs) in resistant cell lines, we generated volcano plots using either A172 (Figure 2A) or LN229 cells (Figure S2) as controls.

In the comparison between A172 and T98G (Figure 2A, top left), *MGMT* is notably upregulated, which is consistent with its established role in TMZ resistance. Additionally, several genes involved in BER, such as *NEIL1* (Nei-Like DNA Glycosylase 1) and *NEIL3* (Nei-Like DNA Glycosylase 3), and double-strand break repair (DSBR), including *ATM* (ATM Serine/Threonine Kinase), *MRE11* (Meiotic Recombination 11 Homolog 1), *RAD54L* (RAD54-like), *XRCC6* (X-Ray Repair Cross Complementing 6) and *PRKDC* (Protein Kinase, DNA-Activated, Catalytic Subunit), are also upregulated, suggesting a broad activation of DNA repair pathways in T98G. Conversely, genes such as *DDB2* (NER), *RAD50* (homologous recombination, HR), *PARP3* (Poly-(ADP-Ribose) Polymerase Family Member 3; BER/HR) and *NEIL2* (Nei Like DNA Glycosylase 2; BER) are significantly downregulated.

When comparing A172 and LN18 cells (Figure 2A, top right), *MGMT*, *NEIL1*, *PRKDC*, *ATM* and *MRE11* were again found to be upregulated, reinforcing the pattern observed in T98G. Interestingly, two key components of NER, such as *DDB2* and *XPA* (Xeroderma Pigmentosum, Complementation Group A), were both downregulated, suggesting a repression of this pathway.

In contrast, U373 cells (Figure 2A, bottom left) revealed a more restricted response, with *MGMT* as the only upregulated gene. Several BER-related genes, including *OGG1* (8-Oxoguanine DNA Glycosylase), *NEIL1*, *PARP3* and *POLB* (DNA Polymerase Beta), as well as *DDB2* (NER), are downregulated, indicating a general suppression of repair activity beyond MGMT-mediated resistance.

Finally, the CCF profile (Figure 2A, bottom right) diverges markedly from the other resistant lines. Most DEGs are downregulated, including *DDB2, XPA, MMS19* (MMS19 homolog, cytosolic iron-sulfur assembly component), and *CDK7* (Cyclin Dependent Kinase 7; NER); *POLL* (DNA Polymerase Lambda)*, POLB*, *PARP3*, and *NEIL2* (BER); *RAD50* (DSBR); and *CCNO* (Cyclin O; cell cycle regulation). Only *MLH3* (MutL Homolog 3), a mismatch repair (MMR) gene, appears upregulated, suggesting a distinct resistance mechanism.

To identify common DEGs in resistant cell lines, we compared upregulated genes (Fold Change > 2 and *p* < 0.05) and downregulated genes (Fold Change < 0.5 and *p* < 0.05) (Figure 2B). Using the LN229 cell line as control (Figure 2B, top), *MLH3* (MMR) was identified as a significantly common upregulated gene and *DDB2* (NER) as a significantly common downregulated gene. On the other hand, when using A172 as control (Figure 2B, bottom), no common upregulated genes were found; however, *DDB2* remained a significantly common downregulated gene. Remarkably, *MGMT* was upregulated in most, but not all, TMZ-resistant cell lines, highlighting variability in its expression across models. In contrast, *DDB2* was consistently downregulated in all resistant lines analysed regardless of the control used, suggesting a potential role of this NER gene in the response to TMZ in GBM cell lines.

For the initial analysis, *PMS1* was used as the normalization reference because it showed the lowest variability among the available reference genes across the samples analyzed. To assess the robustness of the results to the choice of normalization gene, we subsequently repeated the analysis using *GAPDH* as the normalization reference (Figure S3). Although the number and overlap of DEGs varied to some extent between the two normalization strategies, *DDB2* was again identified as the only gene consistently upregulated in both TMZ-sensitive cell lines relative to the TMZ-resistant cell lines. Thus, the identification of *DDB2* as a candidate associated with TMZ responsiveness was not dependent on the normalization strategy.

### 2.3. DDB2 Is Downregulated in TMZ-Resistant GBM Cells and Selectively Induced by TMZ in Sensitive Cells

**Figure 3:**
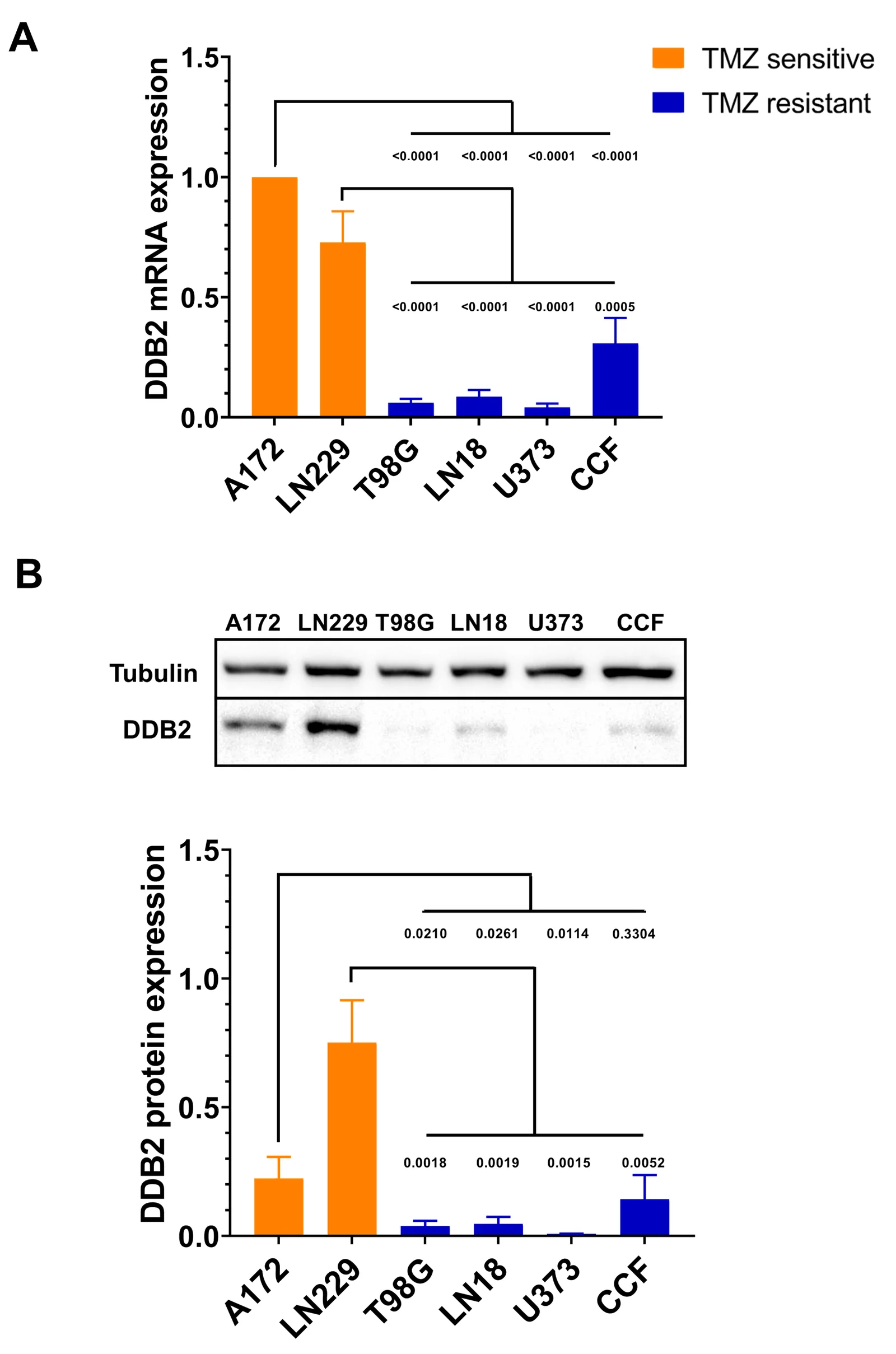
**Reduced DDB2 expression is associated with TMZ resistance in GBM cells.** (**A**) Quantification of DDB2 mRNA levels by RT-qPCR. Relative expression levels were calculated using the 2^−ΔΔCt^ method and normalized to the TMZ-sensitive A172 cell line. Data are the mean ± SD (*n* = 5 independent biological replicates). (**B**) Basal DDB2 protein expression in GBM cell lines. Top: representative immunoblots of DDB2 and tubulin (loading control). Bottom: quantification of DDB2 protein levels normalized to tubulin. Data are the mean ± SD (*n* = 3 independent biological replicates). Statistical significance was assessed using multiple *t*-tests and the corresponding *p*-values are shown for each pairwise comparison.

To validate the differential expression of *DDB2* observed in the transcriptomic analysis, *DDB2* mRNA levels were quantified by RT-qPCR. Consistent with the initial findings, *DDB2* expression was significantly lower in TMZ-resistant cell lines than in each of the TMZ-sensitive models (Figure 3A). Analysis of DDB2 protein abundance further confirmed this pattern, revealing markedly reduced DDB2 levels in TMZ-resistant cells relative to TMZ-sensitive cells (Figure 3B).

Notably, the CCF cell line displayed an intermediate phenotype, with *DDB2* expression levels lower than those observed in TMZ-sensitive cells but higher than those in the remaining TMZ-resistant lines. A similar pattern was observed for *MGMT* expression, which also differed from that of the other resistant models. These findings suggest that the molecular determinants of TMZ response in CCF cells may be distinct from those operating in the other TMZ-resistant cell lines.

**Figure 4:**
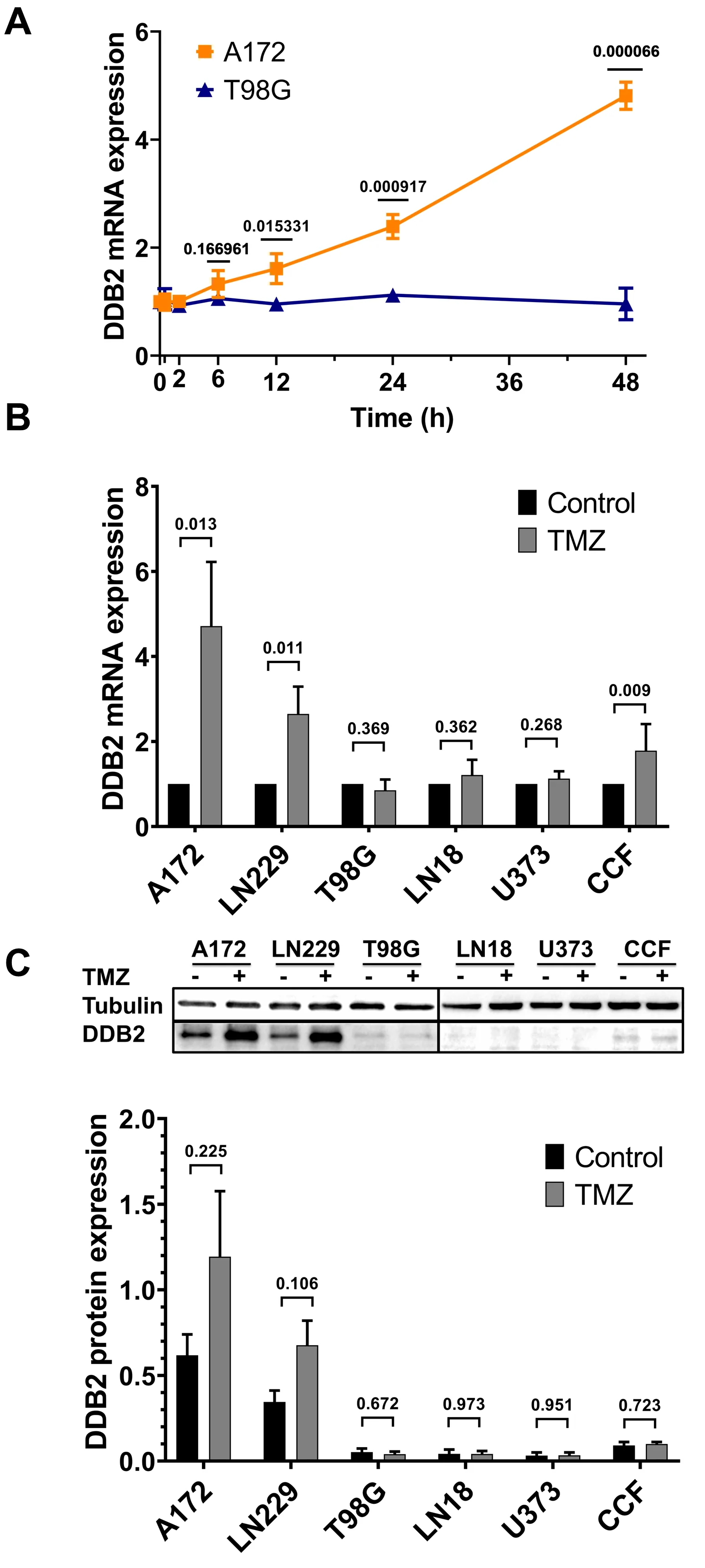
**DDB2 induction following TMZ treatment is restricted to TMZ-sensitive GBM cells.** (**A**) Time-course analysis of *DDB2* mRNA expression following treatment with 100 µM TMZ in TMZ-resistant T98G (blue) and TMZ-sensitive A172 (orange) cell lines. Data are the mean ± SD of relative DDB2 mRNA levels of TMZ-treated cells compared to vehicle-treated controls at each time point (*n* = 3 independent biological replicates). (**B**) Quantification of *DDB2* mRNA expression in six GBM cell lines following 48 h treatment with vehicle control (0.1% DMSO, black) or TMZ (100 μM, gray). Data are the mean ± SD (*n* = 3 independent biological replicates). (**C**) DDB2 protein levels in GBM cell lines under control (−; 0.1% DMSO) and TMZ-treated conditions (+; 100 µM TMZ). Top: representative immunoblots of DDB2 and tubulin (loading control). Bottom: densitometric quantification of DDB2 protein levels normalized to tubulin; data are the mean ± SD (*n* = 3 independent biological replicates). Statistical significance was assessed using multiple *t*-tests and the corresponding *p*-values are shown for each pairwise comparison.

To investigate whether *DDB2* expression is modulated by TMZ treatment, A172 and T98G cells were exposed to TMZ, and *DDB2* expression was monitored over a 48 h period. We found that *DDB2* mRNA levels remained low and unchanged in the TMZ-resistant T98G cell line throughout the treatment. In contrast, *DDB2* expression increased significantly in the TMZ-sensitive A172 cells from 12 h onwards, reaching maximal levels at 48 h following TMZ exposure (Figure 4A).

To determine whether this response was conserved across additional GBM cell lines, *DDB2* expression was assessed 48 h after TMZ treatment, corresponding to the time point of maximal induction observed in A172 cells. TMZ treatment resulted in a significant increase in *DDB2* mRNA levels in the TMZ-sensitive cell lines A172 and LN229, whereas no significant changes were detected in the TMZ-resistant lines (Figure 4B). Consistent with these findings, Western blot analysis revealed increased DDB2 protein abundance following TMZ exposure in sensitive cells, while resistant cells showed little or no induction (Figure 4C). Although the differences in protein levels did not reach statistical significance, the same overall trend was observed across biological replicates (Figure 4C).

Together, these results demonstrate that *DDB2* is selectively induced in response to TMZ in sensitive GBM cell lines, whereas resistant cells fail to upregulate DDB2 following treatment. These findings further support an association between DDB2 expression and TMZ responsiveness in GBM.

### 2.4. DDB2 Depletion Increases TMZ Resistance in A172 GBM Cells

**Figure 5:**
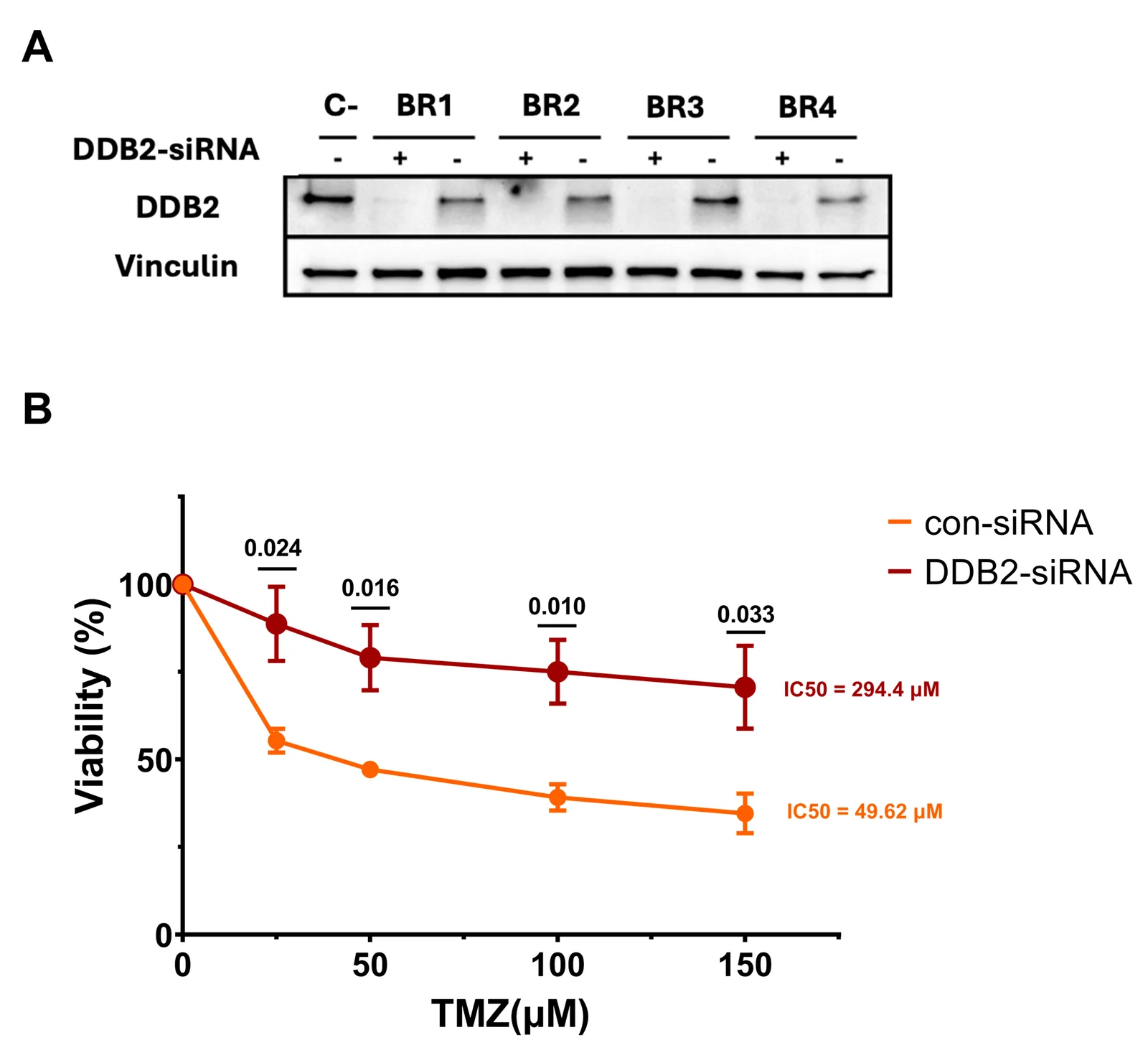
**DDB2 depletion increases TMZ resistance in A172 GBM cells.** (**A**) Representative immunoblot showing DDB2 protein levels in A172 cells transfected with DDB2-targeting siRNA (DDB2-siRNA, +) or a non-targeting control siRNA (con-siRNA, −). Vinculin was used as a loading control. Data are from four independent biological replicates. C-, untransfected cells. (**B**) Cell viability of A172 cells transfected with DDB2-siRNA or con-siRNA following treatment with increasing concentrations of TMZ. The IC50 values are indicated for each survival curve. Data are presented as mean ± SEM (*n* = 4 independent biological replicates). Statistical significance was assessed using multiple *t*-tests and the corresponding *p*-values are shown for each pairwise comparison.

To determine whether DDB2 contributes functionally to the cellular response to TMZ, we depleted DDB2 in the TMZ-sensitive A172 GBM cell line using siRNA-mediated knockdown and subsequently assessed cell viability following TMZ treatment (Figure 5). Efficient reduction in DDB2 expression following siRNA transfection was confirmed prior to drug treatment (Figure 5A). A172 cells were then exposed to increasing concentrations of TMZ, and cell viability was measured after treatment. DDB2 depletion resulted in a significant increase in cell viability in the presence of TMZ compared with control siRNA-transfected cells, indicating that loss of DDB2 reduces the sensitivity of A172 cells to TMZ (Figure 5B). Consistent with this finding, the IC_50_ values for control and DDB2-depleted cells were 49.62 μM and 294.4 μM, respectively, indicative of an increase in TMZ resistance. Thus, depletion of DDB2 is sufficient to attenuate the cytotoxic response of A172 cells to TMZ, supporting a functional role for DDB2 in determining sensitivity to TMZ-induced DNA damage.

### 2.5. Proteomic Profiling Identifies Distinct DNA Damage Response Pathways in TMZ-Sensitive and TMZ-Resistant GBM Cells

To gain mechanistic insight into the differential response to TMZ, we selected A172 and T98G cells as representative models of TMZ-sensitive and TMZ-resistant GBM cells, respectively. Proteomic profiling was performed under basal conditions (DMSO-treated) and following TMZ treatment (100 μM) using a data-independent acquisition (DIA) workflow. A total of 6170 proteins were identified across the four experimental groups.

**Figure 6:**
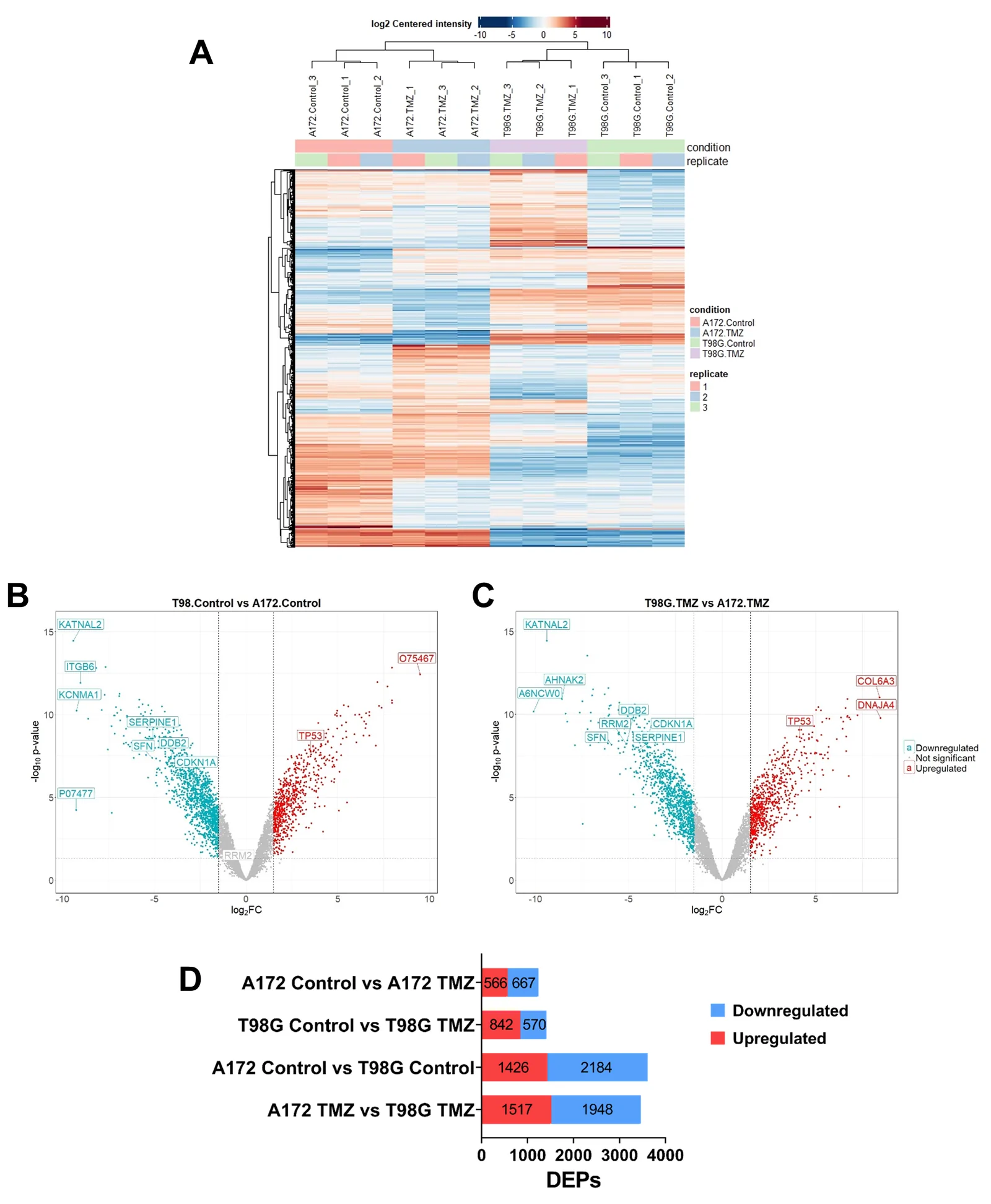
**Proteomic analysis reveals distinct molecular profiles in TMZ-sensitive and TMZ-resistant GBM cells.** (**A**) Heatmap showing the expression patterns of all significantly regulated proteins in A172 and T98G cells following 48 h treatment with vehicle control (DMSO) or TMZ. Each row represents a protein, and each column represents an independent biological replicate. (**B**,**C**) Volcano plots showing differentially expressed proteins (DEPs) between A172 and T98G cells under control conditions (**B**) and following TMZ treatment (**C**). Significantly upregulated proteins (log_2_ fold change > 1.5, *p* < 0.05) are shown in red, whereas significantly downregulated proteins (log_2_ fold change < −1.5, *p* < 0.05) are shown in blue. Proteins not meeting these significance thresholds are shown in gray. (**D**) Number of differentially expressed proteins identified in each pairwise comparison. Upregulated DEPs are shown in red and downregulated DEPs in blue.

Principal component analysis (PCA) and unsupervised hierarchical clustering revealed a clear segregation of samples according to both cell line and treatment condition, indicating high reproducibility between biological replicates and distinct proteomic signatures associated with TMZ sensitivity and resistance (Figure 6A and Figure S4A). Pairwise comparisons identified numerous differentially expressed proteins (DEPs) across experimental conditions (Figure 6B,C and Figure S4B; Tables S12–S19). Notably, the proteomic differences between A172 and T98G cells were substantially greater than those induced by TMZ treatment within each cell line, indicating that intrinsic molecular differences between sensitive and resistant cells dominate the proteomic landscape (Figure 6D).

To identify biological processes associated with these proteomic changes, over-representation analysis (ORA) was performed using Gene Ontology (GO) and KEGG pathway databases. In A172 cells, TMZ treatment was associated with enrichment of processes related to ribosome biogenesis and RNA processing, suggesting extensive remodelling of transcriptional and translational programs in response to DNA damage (Figure S4C). In contrast, TMZ-treated T98G cells showed minimal pathway enrichment, with vesicle-mediated transport to the plasma membrane representing the only significantly enriched GO term, consistent with a limited proteomic response to TMZ (Figure S4C).

**Figure 7:**
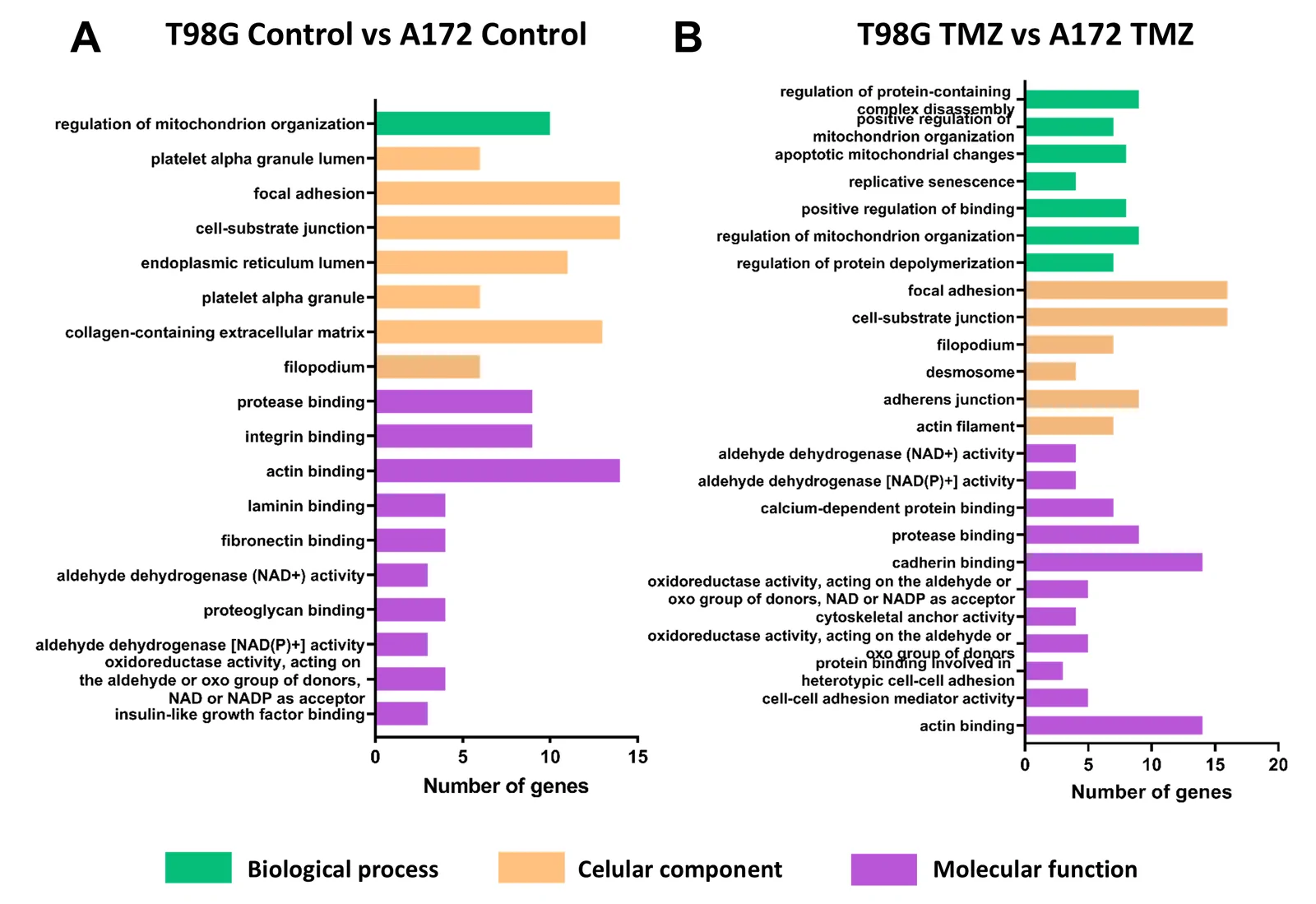
**Gene Ontology (GO) enrichment analysis reveals divergent functional profiles between TMZ-sensitive A172 and TMZ-resistant T98G cells.** (**A**,**B**) GO enrichment analysis of differentially expressed proteins in T98G versus A172 cells under control conditions (**A**) and following TMZ treatment (**B**). Significantly enriched GO terms (*p* < 0.05) are displayed on the *y*-axis, with the number of genes associated with each term shown on the *x*-axis. GO terms are categorized as biological process (green), cellular component (orange), or molecular function (purple). Within each category, terms are ordered by decreasing enrichment significance (increasing *p* value).

Comparisons between A172 and T98G cells yielded a substantially larger number of enriched biological processes (Figure 7). Under basal conditions, enriched GO terms were predominantly related to cell–matrix interactions, cytoskeletal organization, and aldehyde dehydrogenase activity, suggesting inherent differences in cellular architecture and metabolic detoxification pathways between the two models (Figure 7A). Following TMZ treatment, enrichment analysis highlighted pathways associated with mitochondrial organization, apoptotic processes, cell adhesion structures, and oxidoreductase and aldehyde dehydrogenase activities (Figure 7B), indicating that the response to TMZ involves coordinated changes in mitochondrial function, cellular organization and metabolic adaptation.

**Figure 8:**
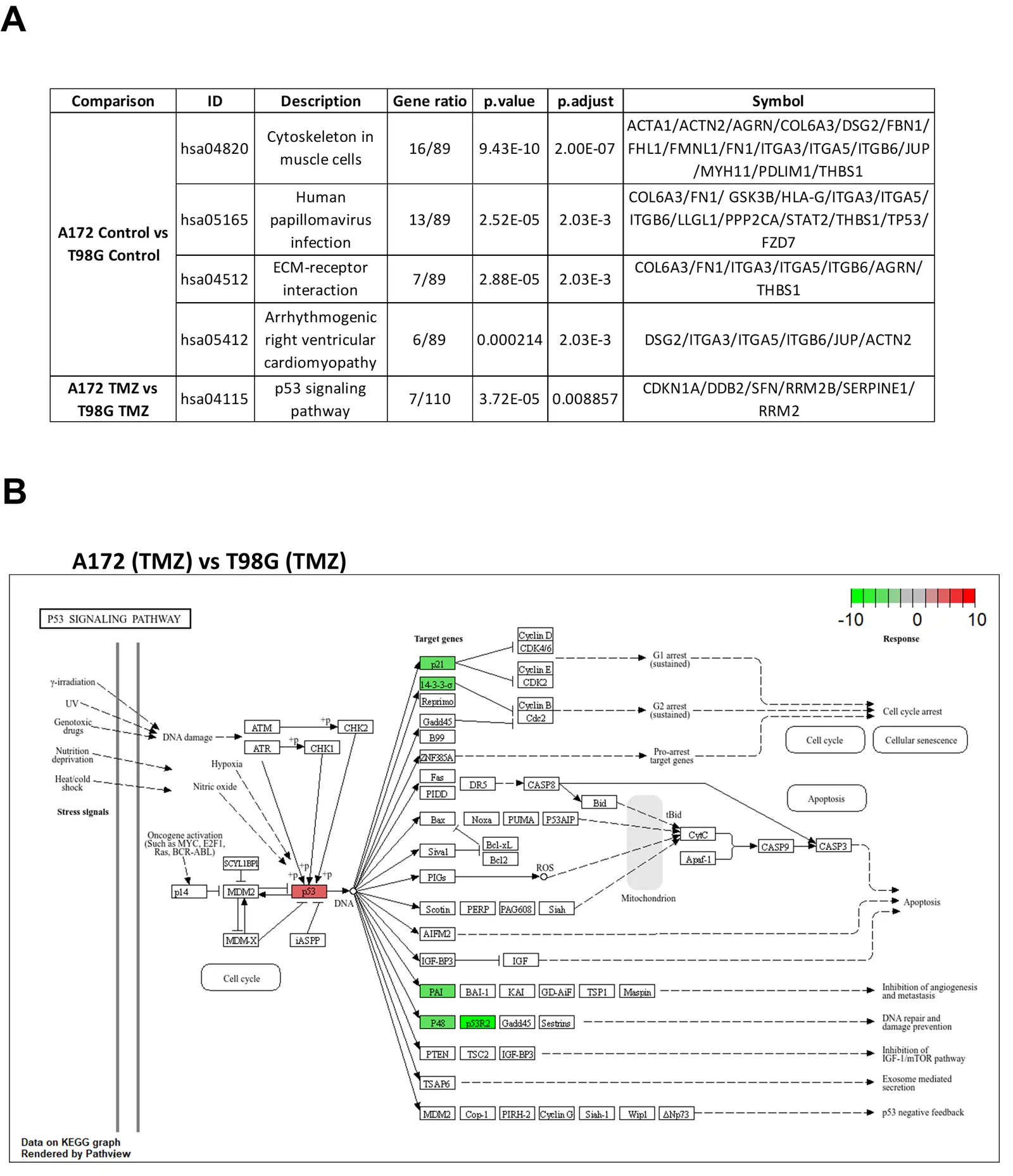
**Enriched KEGG pathways associated with response to TMZ.** (**A**) KEGG pathway enrichment analysis of differentially expressed proteins (DEPs) identified in T98G versus A172 cells under control conditions and following TMZ treatment. (**B**) Mapping of DEPs in T98G versus A172 cells following TMZ treatment onto the KEGG p53 signalling pathway using Pathview (v1.52.0). Nodes represent proteins detected in the proteomic dataset and are colored according to relative abundance, with green indicating decreased abundance and red indicating increased abundance.

KEGG pathway analysis identified significant enrichment exclusively in comparisons between A172 and T98G cells (Figure 8A). Consistent with the GO analysis, pathways related to extracellular matrix interactions and cytoskeletal regulation were enriched under basal conditions. Following TMZ treatment, the p53 signalling pathway emerged as the most significantly enriched pathway, encompassing altered expression of CDKN1A, DDB2, SFN, RRM2B, SERPINE1 and RRM2 (Figure 8A,B). Several of these proteins are established mediators of DNA damage responses, cell-cycle regulation and genome maintenance. Notably, DDB2 was among the proteins contributing to p53 pathway enrichment, further supporting its association with TMZ responsiveness. Collectively, these findings suggest that TMZ-sensitive A172 cells engage a coordinated p53-associated DNA damage response that is largely absent in TMZ-resistant T98G cells.

## 3. Discussion

TMZ resistance remains one of the main barriers to effective GBM treatment and is driven by a complex and heterogeneous set of molecular mechanisms [10]. Among these, DNA repair pathways have emerged as key determinants of therapeutic response because they can counteract TMZ-induced DNA damage and limit its cytotoxic effects. The DNA repair protein MGMT represents the best-established example and is currently used as a predictive biomarker in clinical practice. However, the existence of TMZ-resistant tumours lacking MGMT expression highlights the contribution of additional mechanisms that remain poorly understood [5].

In the present study, we systematically evaluated the expression of genes involved in major DNA repair pathways, including BER, NER, MMR, SSBR and DSBR, across a panel of GBM cell lines with distinct TMZ sensitivity profiles. The panel also included CCF, a human astrocytoma cell line historically classified as a grade IV astrocytoma and more recently used as a model of high-grade glioma/GBM, including in studies of TMZ resistance. We included CCF to broaden the biological diversity of the resistant group, particularly because it provides a TMZ-resistant model with wild-type TP53 and low or undetectable MGMT expression [5,22,23,24,25]. Consistent with previous reports [5,26], MGMT expression alone did not fully explain TMZ responsiveness. In particular, the CCF cell line exhibited a TMZ-resistant phenotype despite displaying MGMT levels comparable to those observed in TMZ-sensitive models. Likewise, several DNA repair genes previously associated with TMZ resistance, including APE1 [17], MPG [16], PARP1 [18], MSH2 [12] and MSH6 [13], failed to consistently discriminate sensitive from resistant cell lines in our experimental setting. These observations reinforce the notion that TMZ response is governed by multiple interacting pathways and suggest that additional determinants remain to be identified.

Among all genes analysed, *DDB2* emerged as the only candidate consistently associated with TMZ sensitivity. *DDB2* expression was markedly reduced in all TMZ-resistant cell lines and significantly increased in TMZ-sensitive cells. Moreover, TMZ exposure induced *DDB2* expression exclusively in sensitive cells, whereas resistant cells failed to upregulate *DDB2* following treatment. Importantly, our functional analysis in A172 cells provides evidence that this association reflects, at least in part, a functional contribution of DDB2 to TMZ responsiveness. siRNA-mediated depletion of DDB2 substantially increased cell viability following TMZ treatment and shifted the TMZ IC_50_ from 49.62 μM in control cells to 294.4 μM in DDB2-depleted cells. Thus, reduced DDB2 expression is sufficient to attenuate the cytotoxic response of A172 cells to TMZ, supporting a functional role for DDB2 in determining TMZ sensitivity. These findings indicate that manipulating DDB2 levels can alter the cellular response to TMZ within the same genetic background. Consequently, the observed relationship cannot be attributed solely to the differences in molecular characteristics that distinguish the TMZ-sensitive and TMZ-resistant cell lines.

DDB2 is best known as a component of the UV-DDB complex, which functions in damage recognition during global genome NER. Through its interaction with DDB1 and the CUL4 ubiquitin ligase complex, DDB2 promotes chromatin remodelling and facilitates access of repair proteins to damaged DNA [19]. In addition, DDB2 collaborates with the CUL4-ROC complex, which ubiquitinates several proteins, including histones H2A, H3 and H4, at sites of DNA damage, thereby facilitating access of other repair factors [27]. Increasing evidence also indicates that DDB2 has functions beyond its canonical role in GG-NER, including regulation of cell proliferation, survival, epithelial-to-mesenchymal transition, migration and invasion [28,29]. Although its role in the response to UV-induced lesions is well established, considerably less is known about how DDB2 influences cellular responses to chemotherapeutic agents. Our identification of DDB2 as a determinant of TMZ responsiveness, together with the functional effect of DDB2 depletion, suggests that DDB2 may contribute to cellular responses to TMZ-induced DNA damage through mechanisms extending beyond its canonical NER function.

Several lines of evidence support the biological relevance of DDB2 in cancer therapy response. Reduced DDB2 expression has been associated with chemoresistance, enhanced stem-like properties and poor clinical outcome in multiple tumour types. In ovarian cancer, DDB2 sensitizes tumour cells to cisplatin-induced apoptosis and suppresses cancer stem cell populations [30,31,32]. Similarly, DDB2 exhibits tumour-suppressive functions in pancreatic ductal adenocarcinoma by limiting epithelial-to-mesenchymal transition and enhancing chemotherapy sensitivity [28]. Notably, transcriptomic studies in gliomas have also identified low DDB2 expression as a feature associated with adverse prognosis [33]. Our findings complement these observations by linking DDB2 expression to TMZ responsiveness in GBM and, importantly, by providing functional evidence that DDB2 depletion reduces TMZ sensitivity. The inclusion of CCF also illustrates the value of examining cell models with diverse molecular backgrounds: despite its distinct historical classification, this cell line displayed the same overall resistant phenotype and contributed to the consistent identification of reduced DDB2 expression across the resistant group.

The mechanisms linking *DDB2* to TMZ sensitivity remain speculative, but several possibilities can be considered. One potential explanation involves a functional interaction between DDB2 and the BER machinery responsible for processing N3-meA and N7-meG lesions, which constitute the majority of TMZ-induced DNA adducts. Previous studies have demonstrated that DDB2 binds abasic sites, enhances MPG glycosylase activity, stimulates APE1-mediated cleavage and promotes chromatin accessibility at sites of DNA damage [34,35,36,37]. Importantly, DDB2-deficient cells display increased resistance to the alkylating agent methyl methanesulfonate [36], suggesting that DDB2 may facilitate the generation of cytotoxic BER intermediates following alkylation damage. Because BER intermediates such as abasic sites and single-strand breaks can themselves contribute substantially to cytotoxicity [38], reduced DDB2 expression could attenuate this process and thereby promote cellular survival after TMZ exposure [39]. Our observation that DDB2 depletion increases the TMZ IC_50_ in A172 cells is consistent with this model, although the present study does not establish whether the effect of DDB2 depletion is mediated specifically through BER. The reported MMS resistance of DDB2-deficient cells [36] is nevertheless consistent with the possibility that DDB2 influences cellular responses to alkylation-induced damage more broadly. Establishing whether this is the case will require direct comparative analyses with additional alkylating agents. Additional mechanistic experiments will be required to determine whether altered processing of TMZ-induced lesions or downstream BER intermediates accounts for the phenotype.

An additional, non-mutually exclusive explanation relates to the role of DDB2 in regulating cell-fate decisions following DNA damage. Beyond DNA repair, DDB2 has emerged as a multifunctional regulator of apoptosis, senescence and tumour suppression [40]. Notably, DDB2 and CDKN1A (p21) are transcriptional targets of p53, and DDB2 has been reported to facilitate apoptosis by limiting p21 accumulation after genotoxic stress [29]. In this context, our proteomic analyses are particularly informative. Following TMZ treatment, the p53 signalling pathway was selectively enriched in A172 cells and included altered expression of DDB2, CDKN1A, SFN, RRM2B and other canonical p53-responsive proteins. By contrast, this coordinated response was largely absent in T98G cells. These observations are consistent with the possibility that impaired p53 signalling contributes to the reduced induction of DDB2 following TMZ treatment in some resistant cells. However, the relationship between DDB2 and p53 status does not appear to be sufficient to explain our findings. In particular, CCF cells harbour wild-type TP53 [22,23,25] (Supplementary Table S20) yet display a TMZ-resistant phenotype and, like the other resistant cell lines, fail to induce DDB2 expression following TMZ treatment. Thus, differences in p53 status alone cannot account for the association between DDB2 expression and TMZ responsiveness, suggesting that additional mechanisms regulate DDB2 expression or activity in response to TMZ. Moreover, our loss-of-function experiment in A172 cells provides direct functional evidence that DDB2 itself contributes to TMZ responsiveness, as DDB2 depletion markedly reduced TMZ sensitivity within the same cellular background. Taken together, these findings support a model in which p53 signalling may contribute to the regulation of DDB2 following TMZ exposure, while DDB2 additionally plays a functional role in determining the cellular response to TMZ.

Overall, our study identifies DDB2 through an unbiased comparative analysis of DNA repair gene expression across GBM cell lines with distinct TMZ responses and provides functional evidence that DDB2 contributes to TMZ sensitivity. While CCF has a distinct historical classification, its inclusion broadened the molecular diversity of the resistant group and did not alter the identification of DDB2 as a common feature of the resistant models. Our findings are compatible with the established involvement of DDB2 in DNA repair and cell-fate regulation, but the precise molecular mechanism by which DDB2 influences the response to TMZ remains to be established. Further studies, including complementary gain-of-function and rescue experiments, validation in additional TMZ-resistant and TMZ-sensitive models, and analysis of patient-derived systems and clinical cohorts, will be important to define the generality and translational relevance of this finding. In particular, comparative studies with additional alkylating agents will be needed to determine whether the role of DDB2 observed in TMZ responsiveness is specific to TMZ or reflects a broader function in the cellular response to alkylation-induced DNA damage.

## 4. Materials and Methods

### 4.1. Cell Lines and Culture Conditions

Six human glioblastoma (GBM) cell lines were obtained from the American Type Culture Collection (ATCC, Manassas, VA, USA) and Sigma-Aldrich (St. Louis, MO, USA): A172 (CRL-1620), LN229 (CRL-2611), T98G (CRL-1690), LN18 (CRL-2610), U373 (U373 MG; 08061901), and CCF-STTG1 (CRL-1718). A172, LN229, and LN18 cells were maintained in Dulbecco’s Modified Eagle Medium (DMEM; Sigma, St. Louis, MO, USA). T98G and U373 cells were cultured in Minimum Essential Medium (MEM; Biowest, Nuaillé, France), whereas CCF-STTG1 cells were grown in Roswell Park Memorial Institute medium (RPMI; Biowest). All media were supplemented with 10% fetal bovine serum (FBS; Biowest), 100 U/mL penicillin (Merck, Darmstadt, Germany), 100 μg/mL streptomycin (Merck), and 1 mM sodium pyruvate (Gibco, Waltham, MA, USA). MEM was additionally supplemented with 1% non-essential amino acids (Sigma-Aldrich), while RPMI was supplemented with 1% L-glutamine (Biowest). Cells were cultured at 37 °C in a humidified incubator with 5% CO_2_. Upon reaching 80–90% confluence, cells were detached using trypsin, counted, collected by centrifugation, and seeded at densities appropriate for each experiment. All cell lines were tested for mycoplasma contamination and confirmed to be negative.

### 4.2. siRNA-Mediated Depletion of DDB2

For gene silencing experiments, 0.25 × 10^6^ cells were seeded per well in 6-well plates and allowed to adhere for 24 h prior to siRNA transfection. Cells were transfected with 25 nM siRNA (Dharmacon, Supplementary Table S21) using 7.5 μL Lipofectamine RNAiMAX Transfection Reagent (Invitrogen, Waltham, MA, USA) diluted in Opti-MEM (Gibco), following the manufacturer’s instructions. After 24 h, serum-free culture medium was added to each well at a 1:1 ratio. Cells were harvested 48 h post-transfection for subsequent experiments.

### 4.3. Cell Survival Assays

#### 4.3.1. Cell Proliferation Assay

Cells were seeded at a density of 10,000 cells per well in 12-well plates using the appropriate culture medium for each cell line. Cells were treated with temozolomide (TMZ; Sigma-Aldrich) at the indicated concentrations. After 7 days of treatment, cells were fixed with absolute ethanol and stained with crystal violet solution (0.05% *w/v* crystal violet, 1% formaldehyde and 1% methanol). Plates were imaged using the ChemiDoc MP Imaging System (Bio-Rad, Hercules, CA, USA). Cell proliferation was quantified by measuring the integrated signal intensity (Volume Int) using Image Lab software (version 1.54, Bio-Rad).

#### 4.3.2. Cell Viability Assay

Cells were seeded at a density of 7000 cells per well in 24-well plates using the appropriate culture medium for each cell line. Cells were treated with temozolomide (TMZ; Sigma-Aldrich) at the indicated concentrations. After 5 days of treatment, culture medium was removed and replaced with a 1:10 dilution of WST-1 reagent (Roche, Basel, Switzerland) prepared in the corresponding culture medium. Following incubation for 30 min at 37 °C, supernatants were collected and transferred to 96-well plates. Absorbance was measured at 595 nm using a microplate reader (PowerWave HD, BioTek, Winooski, VT, USA).

Half-maximal inhibitory concentration (IC_50_) values were determined by nonlinear regression analysis using a four-parameter logistic (4PL) dose–response model in GraphPad Prism 9 (GraphPad Software, San Diego, CA, USA).

### 4.4. RNA Extraction, RT^2^ Profiler PCR Array and RT-qPCR

Total RNA was extracted using the RNeasy Mini Kit (Qiagen, Germantown, MD, USA), according to the manufacturer’s instructions. RNA quality was assessed using High Sensitivity RNA ScreenTape^®^ analysis and only samples with an RNA integrity number (RIN) up to 9 were included in subsequent analyses.

For RT^2^ Profiler PCR Array analysis, cDNA was synthesized from 0.5 μg of total RNA using the RT^2^ First Strand Kit (Qiagen) following the manufacturer’s protocol. Expression profiling of 84 DNA repair-related genes was performed using the Human DNA Repair RT^2^ Profiler PCR Array (PAHS-042ZA, Qiagen) (Figure S1) on a CFX96 Real-Time PCR Detection System (Bio-Rad). Data were analysed using Qiagen GeneGlobe software (https://geneglobe.qiagen.com), VolcaNoseR v1.0.3, and Venny 2.0. Cycle threshold (Ct) values were used to calculate relative gene expression changes using the 2^−ΔΔCt^ method [41]. Data were normalized to *PMS1*, selected using the “Automatic Selection from Full Panel” option, which identifies the reference gene set with the minimal variation in geometric mean across samples.

For reverse transcription quantitative PCR (RT-qPCR) validation, cDNA was synthesized from 1 μg of total RNA using the qScript cDNA Synthesis Kit (Quanta Biosciences, Beverly, MA, USA) in a final reaction volume of 20 μL. cDNA was subsequently diluted 1:5 in nuclease-free water. RT-qPCR reactions were prepared using 2 μL of diluted cDNA, 0.5 μM of each primer (Table S1), and 1× NZYSpeedy Green Master Mix (NZYtech, Lisboa, Portugal). Amplification was performed on a CFX96 Real-Time PCR Detection System (Bio-Rad), and data were analysed using CFX Manager software (version 3.1, Bio-Rad). Relative gene expression was calculated using the 2^−ΔΔCt^ method [41] with *GAPDH* as the reference gene.

### 4.5. Immunoblotting Analysis

To obtain whole-cell protein extracts, cell pellets were lysed in RIPA buffer (Sigma-Aldrich) supplemented with Protease Inhibitor Mix M (Serva, Heidelberg, Germany). Lysates were centrifuged at 10,000× *g* for 30 min at 4 °C, and the supernatants were collected and stored at −80 °C until use. Protein concentration was determined using the Pierce BCA Protein Assay Kit according to the manufacturer’s instructions. Absorbance was measured at 562 nm using a microplate reader (PowerWave HD, BioTek). Equal amounts of protein (15 μg) were separated by 12% SDS-PAGE and transferred onto nitrocellulose membranes (Bio-Rad). Membranes were incubated with mouse monoclonal anti-DDB2 antibodies (1:1000; sc-81246, Santa Cruz Biotechnology, Dallas, TX, USA), and mouse monoclonal anti-α-tubulin (T5168, Sigma-Aldrich) and anti-vinculin (V9131, Sigma-Aldrich) antibodies were used as loading controls, as indicated. Subsequently, membranes were incubated with HRP-conjugated horse anti-mouse IgG secondary antibodies (7076P2, Cell Signaling Technology, Danvers, MA, USA). ECL Prime Western blotting Detection Reagent (Amersham, UK) was used for α-tubulin detection, and Lumigen ECL Ultra was used for DDB2 detection (Lumigen, Southfield, MI, USA), and signals were visualised using the ChemiDoc MP Imaging System (Bio-Rad). Band intensities were quantified using Image Lab software (Bio-Rad).

### 4.6. Data-Independent Acquisition (DIA) Proteomic Analysis

DIA-based proteomic analysis was performed in three biological replicates of TMZ-treated (100 μM) and untreated (0.1% DMSO) A172 and T98G GBM cells. Protein cell extracts were obtained as described above, and supernatants were stored at −80 °C until proteomic analysis. After thawing, proteins were precipitated using a methanol-chloroform protocol, sonicated, and quantified using the Qubit Protein Assay Kit (Thermo Fisher Scientific, Waltham, MA, USA) according to the manufacturer’s instructions. Protein digestion was performed using the iST Kit (PreOmics, Martinsried, Germany) following the manufacturer’s protocol. Samples were analysed by liquid chromatography-tandem mass spectrometry (LC-MS/MS) using DIA-PASEF acquisition mode on the Evosep One-timsTOF Flex Clinical Proteomics Platform (IMSMI). Following MS data acquisition, a total of 6170 proteins were identified and quantified at a false discovery rate (FDR) of 5%.

### 4.7. Proteomic Data Processing and Functional Enrichment Analysis

Proteomic data were analysed in R (v4.4.1) using the DEP package (v1.26.0) to assess differential protein expression across experimental conditions. Prior to statistical analysis, data were normalized using variance stabilizing normalization (VSN) to minimize systematic variability between samples. Missing values, assumed to originate primarily from low-abundance proteins, were imputed using a left-censored distribution implemented through the manual imputation function in DEP. Principal component analysis (PCA) was performed to assess global variance structure and evaluate sample clustering within and across experimental groups. Proteins were considered differentially expressed when presenting an adjusted *p* value < 0.05 and an absolute log_2_ fold change ≥ 1.5. Functional enrichment analysis of differentially expressed proteins was performed using Over-Representation Analysis (ORA) of Gene Ontology (GO) terms and Kyoto Encyclopedia of Genes and Genomes (KEGG) pathways with the clusterProfiler package (v4.12.6). Multiple testing correction was performed using the Benjamini–Hochberg method, with an adjusted *p* value threshold of 0.05.

### 4.8. Quantification and Statistical Analysis

Statistical analyses were performed using GraphPad Prism 9. The statistical tests applied, sample sizes, definitions of significance, and measures of central tendency and data dispersion are specified in the corresponding tables and figure legends.

## 5. Conclusions

We identify DDB2 expression as a robust molecular feature distinguishing TMZ-sensitive from TMZ-resistant GBM cells. The differential basal expression of DDB2, together with its selective induction following TMZ treatment, identifies DDB2 as a candidate biomarker of TMZ responsiveness. Importantly, we found that DDB2 contributes functionally to TMZ sensitivity, as DDB2 depletion substantially reduced the response of A172 GBM cells to TMZ. These findings support a role for DDB2 in modulating the cellular response to TMZ-induced DNA damage and provide a rationale for further investigating DDB2 as a potential therapeutic target for TMZ resistance, while additional mechanistic studies and validation in other GBM models will be required to establish its broader therapeutic relevance.

## Data Availability

The original contributions presented in this study are included in the article/Supplementary Material. Further inquiries can be directed to the corresponding author.

## Supplementary Materials

The following supporting information can be downloaded at: https://www.mdpi.com/article/10.3390/ijms27208941/s1. References [42,43] are cited in Supplementary Materials.
