# Supplementary material for: Reduced DDB2 Expression Is Associated with Temozolomide Resistance in Glioblastoma Cells
Source: Int J Mol Sci. 2026 Oct 9;27(20):8941. doi: 10.3390/ijms27208941 (PMC13704723; doi:10.3390/ijms27208941)
Supplement: Supplementary file 1 [file ijms-27-08941-s001.zip › ijms-4456213-supplementary.pdf]

Table S1. Primer sequences

Primer	Sequence
**GAPDH Fw**	5'-TGTCCCCACTGCCAACGTGTCA-3'
**GAPDH Rv**	3'- AGCGTCAAAGGTGGAGGAGTGGGT-5'
**DDB2 Fw**	5'- CCAACCAGTTTTACGCCTCCTC – 3'
**DDB2 Rv**	3'- TGTCTCCTGTGACCACCATTCG -5'

Table S2. Summary of two-way ANOVA analysis for crystal violet proliferation assays

Source of variation	df	F	p-value
**TMZ doses**	5	54.66	**<0.0001**
**Cell line**	5	12.00	**0.0002**
**TMZ doses × Cell line**	25	7.417	**<0.0001**
**Residuals**	60	—	—

Table S3. Dunnett's post hoc comparisons of TMZ-treated groups versus control conditions in crystal violet proliferation assays

Comparison/Cell line		A172	LN229	T98G	LN18	U373	CCF
**0 µM vs 5 µM**	Mean difference	15.63	15.93	1.844	2.362	2.524	0.9202
	95% CI	6.324 to 24.94	-10.66 to 42.53	-10.79 to 14.48	-12.04 to 16.76	-14.77 to 19.82	-3.954 to 5.795
	Adjusted p-value	**0.0182**	0.1297	0.7831	0.7265	0.7831	0.6512
**0 µM vs 10 µM**	Mean difference	29.61	28.33	11.53	7.937	1.838	2.873
	95% CI	26.55 to 32.67	-4.679 to 61.34	-64.22 to 87.29	-4.769 to 20.64	-7.278 to 10.95	-5.480 to 11.23
	Adjusted p-value	**0.0006**	0.0669	0.7634	0.1204	0.6141	0.3225
**0 µM vs 20 µM**	Mean difference	39.45	45.71	11.19	12.07	8.765	5.18
	95% CI	21.88 to 57.02	9.088 to 82.33	-65.52 to 87.91	-18.98 to 43.12	-23.01 to 40.54	0.6225 to 9.738
	Adjusted p-value	**0.0103**	**0.0326**	0.7831	0.2683	0.4356	**0.0391**
**0 µM vs 50 µM**	Mean difference	41.05	51.98	8.236	4.361	11.38	5.361
	95% CI	29.67 to 52.42	32.13 to 71.83	-42.87 to 59.34	-12.89 to 21.61	-42.54 to 65.30	1.698 to 9.025
	Adjusted p-value	**0.004**	**0.0076**	0.7355	0.4841	0.5879	**0.0239**
**0 µM vs 100 µM**	Mean difference	44.14	56.27	12.97	10.49	15.91	7.13
	95% CI	29.60 to 58.68	33.58 to 78.95	-75.91 to 101.8	-8.379 to 29.36	-43.11 to 74.93	-3.000 to 17.26
	Adjusted p-value	**0.0057**	**0.0084**	0.7831	0.1481	0.4481	0.0969

Table S4. Tukey's post hoc multiple-comparisons test between GBM cell lines in crystal violet proliferation assays

Comparison/Treatment		0 µM	5 µM	10 µM	20 µM	50 µM	100 µM
**A172 vs. LN229**	Mean difference	0	0.2991	-1.277	6.259	10.93	12.13
	95% CI		-26.99 to 27.58	-40.63 to 38.07	-28.52 to 41.04	-7.248 to 29.11	-8.352 to 32.61
	Adjusted p-value		>0.9999	0.9995	0.8726	0.1874	0.2046
**A172 vs. T98G**	Mean difference	0	-13.79	-18.07	-28.26	-32.81	-31.17
	95% CI		-25.18 to -2.401	-109.5 to 73.37	-113.6 to 57.13	-89.93 to 24.31	-134.0 to 71.62
	Adjusted p-value		**0.027**	0.6525	0.3937	0.1526	0.4218
**A172 vs. LN18**	Mean difference	0	-13.27	-21.67	-27.38	-36.69	-33.65
	95% CI		-26.26 to -0.2768	-35.71 to -7.633	-55.87 to 1.120	-52.23 to -21.15	-50.72 to -16.58
	Adjusted p-value		**0.047**	**0.0196**	0.0559	**0.0031**	**0.0049**
**A172 vs. U373**	Mean difference	0	-13.11	-27.77	-30.69	-29.67	-28.23
	95% CI		-29.13 to 2.913	-37.21 to -18.32	-59.97 to -1.402	-90.36 to 31.03	-93.19 to 36.73
	Adjusted p-value		0.0855	**0.0042**	**0.0439**	0.204	0.2588
**A172 vs. CCF**	Mean difference	0	-14.71	-26.73	-34.27	-35.68	-37.01
	95% CI		-23.38 to -6.041	-35.21 to -18.25	-53.45 to -15.09	-47.58 to -23.79	-50.09 to -23.93
	Adjusted p-value		**0.0112**	**0.0034**	**0.0139**	**0.0042**	**0.0015**
**LN229 vs. T98G**	Mean difference	0	-14.09	-16.8	-34.51	-43.74	-43.3
	95% CI		-39.41 to 11.23	-90.46 to 56.86	-107.5 to 38.44	-94.88 to 7.396	-140.5 to 53.95
	Adjusted p-value		0.2039	0.7376	0.2842	0.0724	0.2501
**LN229 vs. LN18**	Mean difference	0	-13.57	-20.39	-33.64	-47.62	-45.78
	95% CI		-38.17 to 11.04	-53.49 to 12.71	-67.34 to 0.06622	-66.01 to -29.23	-66.56 to -25.00
	Adjusted p-value		0.2214	0.1508	0.0503	**0.0016**	**0.0031**
**LN229 vs. U373**	Mean difference	0	-13.41	-26.49	-36.94	-40.6	-40.36
	95% CI		-37.39 to 10.58	-62.10 to 9.121	-70.85 to -3.035	-95.25 to 14.06	-99.57 to 18.85
	Adjusted p-value		0.2351	0.0941	**0.0376**	0.099	0.1211
**LN229 vs. CCF**	Mean difference	0	-15.01	-25.46	-40.53	-46.62	-49.14
	95% CI		-45.44 to 15.42	-61.63 to 10.71	-83.67 to 2.616	-69.31 to -23.92	-71.06 to -27.21
	Adjusted p-value		0.1967	0.1042	0.0566	**0.0113**	**0.0059**
**T98G vs. LN18**	Mean difference	0	0.5181	-3.595	0.8778	-3.875	-2.479
	95% CI		-12.86 to 13.90	-91.02 to 83.83	-75.10 to 76.86	-56.76 to 49.01	-102.4 to 97.49
	Adjusted p-value		>0.9999	0.9987	>0.9999	0.9921	>0.9999
**T98G vs. U373**	Mean difference	0	0.6796	-9.695	-2.43	3.144	2.942
	95% CI		-14.90 to 16.26	-99.09 to 79.70	-77.97 to 73.11	-48.34 to 54.63	-77.11 to 83.00
	Adjusted p-value		0.9999	0.9259	0.9999	0.9995	>0.9999
**T98G**	Mean difference	0	-0.9241	-8.659	-6.014	-2.875	-5.838

**vs. CCF**	95% CI		-13.59 to 11.74	-98.41 to 81.10	-98.30 to 86.27	-64.18 to 58.43	-111.0 to 99.31
	Adjusted p-value		0.9939	0.9489	0.9875	0.997	0.9943
**LN18 vs. U373**	Mean difference	0	0.1615	-6.099	-3.307	7.019	5.422
	95% CI		-15.69 to 16.01	-17.53 to 5.335	-34.06 to 27.44	-49.44 to 63.48	-56.39 to 67.24
	Adjusted p-value		>0.9999	0.2727	0.9927	0.9341	0.9819
**LN18 vs. CCF**	Mean difference	0	-1.442	-5.064	-6.892	1.001	-3.359
	95% CI		-16.33 to 13.45	-16.52 to 6.388	-43.11 to 29.32	-18.40 to 20.40	-20.85 to 14.13
	Adjusted p-value		0.9753	0.3818	0.6988	0.997	0.8666
**U373 vs. CCF**	Mean difference	0	-1.604	1.035	-3.585	-6.018	-8.781
	95% CI		-20.19 to 16.99	-7.556 to 9.626	-40.71 to 33.54	-70.75 to 58.71	-76.75 to 59.19
	Adjusted p-value		0.9801	0.9878	0.9521	0.9521	0.8858

Table S5. Summary of two-way ANOVA analysis for WST-1 viability assays

**Source of variation**	**df**	**F**	**p-value**
**TMZ doses**	4	118.9	**<0.0001**
**Cell line**	5	41.46	**<0.0001**
**TMZ doses × Cell line**	20	18.61	**<0.0001**
**Residuals**	48	—	—

Table S6. Dunnett's post hoc comparisons of TMZ-treated groups versus control conditions in WST-1 viability assays

**Comparison/Cell line**		**A172**	**LN229**	**T98G**	**LN18**	**U373**	**CCF**
**0 µM vs 25 µM**	Mean difference	65.23	69.56	6.315	10.13	13.08	8.466
	95% CI	14.84 to 115.6	55.40 to 83.72	-29.88 to 42.51	-19.65 to 39.91	-25.63 to 51.79	-46.67 to 63.61
	Adjusted p-value	**0.0304**	**0.0022**	0.6799	0.324	0.3273	0.7434
**0 µM vs 50 µM**	Mean difference	63.91	74.11	6.174	13.4	19.42	12.46
	95% CI	43.87 to 83.95	62.47 to 85.76	-20.45 to 32.79	-18.44 to 45.25	-24.22 to 63.07	-43.57 to 68.50
	Adjusted p-value	**0.0051**	**0.0013**	0.5239	0.2348	0.2147	0.5474
**0 µM vs 100 µM**	Mean difference	72.11	80.13	8.302	15.89	21.67	8.466
	95% CI	65.69 to 78.52	75.34 to 84.93	-18.79 to 35.40	-8.446 to 40.23	-6.770 to 50.12	-46.67 to 63.61
	Adjusted p-value	**0.0004**	**0.0002**	0.3745	0.1108	0.0835	0.7434
**0 µM vs 150 µM**	Mean difference	78.14	84.41	9.132	7.758	31.35	12.92
	95% CI	40.27 to 116.0	53.83 to 115.0	-20.91 to 39.17	-17.52 to 33.03	-14.97 to 77.66	-45.66 to 71.49
	Adjusted p-value	**0.0122**	**0.0068**	0.3785	0.3737	0.1039	0.5521

Table S7. Tukey's post hoc multiple-comparisons test between GBM cell lines in WST-1 viability assays

Comparison/Treatment		0 µM	25 µM	50 µM	100 µM	150 µM
**A172 vs. LN229**	Mean difference	0	4.33	10.2	8.027	6.262
	95% CI		-52.69 to 61.35	-9.063 to 29.47	1.943 to 14.11	-30.03 to 42.56
	Adjusted p-value		0.9881	0.2504	**0.0203**	0.9438
**A172 vs. T98G**	Mean difference	0	-58.91	-57.74	-63.8	-69.01
	95% CI		-106.6 to -11.20	-83.01 to -32.46	-95.38 to -32.23	-105.2 to -32.82
	Adjusted p-value		**0.0255**	**0.003**	**0.0109**	**0.0054**
**A172 vs. LN18**	Mean difference	0	-55.1	-50.51	-56.21	-70.39
	95% CI		-103.4 to -6.774	-80.81 to -20.20	-84.08 to -28.34	-106.3 to -34.50
	Adjusted p-value		**0.0342**	**0.0103**	**0.0106**	**0.0057**
**A172 vs. U373**	Mean difference	0	-52.15	-44.49	-50.43	-46.8
	95% CI		-100.1 to -4.211	-88.56 to -0.4091	-83.82 to -17.04	-91.29 to -2.306
	Adjusted p-value		**0.0382**	**0.0488**	**0.0207**	**0.0424**
**A172 vs. CCF**	Mean difference	0	-56.76	-51.45	-63.64	-65.23
	95% CI		-111.4 to -2.143	-111.6 to 8.739	-132.2 to 4.939	-120.8 to -9.621
	Adjusted p-value		**0.044**	0.0718	0.0579	**0.0308**
**LN229 vs. T98G**	Mean difference	0	-63.24	-67.94	-71.83	-75.27
	95% CI		-101.3 to -25.21	-95.15 to -40.73	-104.6 to -39.10	-106.5 to -44.06
	Adjusted p-value		**0.0142**	**0.0045**	**0.0101**	**0.002**
**LN229 vs. LN18**	Mean difference	0	-59.43	-60.71	-64.24	-76.65
	95% CI		-89.25 to -29.61	-94.73 to -26.69	-93.31 to -35.17	-106.1 to -47.21
	Adjusted p-value		**0.0077**	**0.0123**	**0.0097**	**0.0016**
**LN229 vs. U373**	Mean difference	0	-56.48	-54.69	-58.46	-53.06
	95% CI		-97.84 to -15.13	-104.5 to -4.832	-92.98 to -23.94	-96.97 to -9.149
	Adjusted p-value		**0.0237**	**0.0409**	**0.0169**	**0.028**
**LN229 vs. CCF**	Mean difference	0	-61.09	-61.65	-71.67	-71.49
	95% CI		-124.5 to 2.322	-128.2 to 4.851	-141.0 to -2.377	-128.9 to -14.05
	Adjusted p-value		0.0542	0.0585	**0.0468**	**0.0273**
**T98G vs. LN18**	Mean difference	0	3.815	7.229	7.592	-1.374
	95% CI		-30.99 to 38.62	-23.50 to 37.96	-19.09 to 34.27	-30.41 to 27.66
	Adjusted p-value		0.9912	0.8467	0.75	0.9998
**T98G vs. U373**	Mean difference	0	6.761	13.25	13.37	22.21
	95% CI		-31.92 to 45.44	-28.42 to 54.92	-15.26 to 42.01	-21.75 to 66.18
	Adjusted p-value		0.946	0.6054	0.3867	0.2924
**T98G**	Mean difference	0	2.151	6.289	0.1636	3.785

**vs. CCF**	95% CI		-50.14 to 54.44	-49.85 to 62.42	-54.64 to 54.97	-53.88 to 61.45
	Adjusted p-value		0.9999	0.9753	>0.9999	0.9979
**LN18 vs. U373**	Mean difference	0	2.946	6.021	5.781	23.59
	95% CI		-33.89 to 39.78	-35.33 to 47.37	-21.83 to 33.39	-21.44 to 68.61
	Adjusted p-value		0.9977	0.967	0.8947	0.2481
**LN18 vs. CCF**	Mean difference	0	-1.664	-0.9397	-7.429	5.159
	95% CI		-55.37 to 52.04	-55.01 to 53.13	-63.66 to 48.80	-54.91 to 65.23
	Adjusted p-value		>0.9999	>0.9999	0.9477	0.99
**U373 vs. CCF**	Mean difference	0	-4.61	-6.96	-13.21	-18.43
	95% CI		-56.80 to 47.58	-60.36 to 46.44	-67.42 to 41.00	-74.37 to 37.51
	Adjusted p-value		0.9958	0.9805	0.7392	0.6354

Table S8. Temozolomide IC_{50} values (μM) in cell proliferation assays

		A172	LN229	T98G	LN18	U373	CCF
Best-fit values	IC_{50}	61.15	38.51	523.4	704.4	453.1	1060.0
	$logIC_{50}$	1.786	1.586	2.719	2.848	2.656	3.025
95% CI	IC_{50}	40.30 – 94.98	27.65 – 54.12	231.7 – 8355.0	427.6 – 1634.0	283.6 – 917.3	814.2 – 1491.0
	$logIC_{50}$	1.605 – 1.978	1.442 – 1.733	2.365 – 3.922	2.631 – 3.213	2.453 – 2.963	2.911 – 3.173

Table S9. Temozolomide IC_{50} values (μM) in cell viability assays

		A172	LN229	T98G	LN18	U373	CCF
Best-fit values	IC_{50}	23.17	15.24	1188.0	792.1	301.6	832.5
	$logIC_{50}$	1.365	1.183	3.075	2.899	2.479	2.92
95% CI	IC_{50}	15.44 - 33.27	11.54 - 19.55	712.8 – 2916.0	475.7 – 1824.0	218.1 - 442.0	417.1 – 4060.0
	$logIC_{50}$	1.189 - 1.522	1.062 - 1.291	2.853 - 3.465	2.677 - 3.261	2.339 - 2.645	2.620 - 3.609

Table S10. Differentially expressed genes in GBM cell lines relative to the TMZ-sensitive A172 cell line

Cell line	Name	Change	Fold change (log2)	p-value (-log10)	Manhattan distance
LN229	MLH3	Decreased	-3.64385619	2.54928912	6.19314531
	PARP3	Decreased	-2	2.19477109	4.19477109
	NEIL2	Decreased	-1.55639335	2.15577142	3.71216477
	RAD50	Decreased	-1.47393119	2.09441997	3.56835116
	POLB	Decreased	-1.39592868	1.85349987	3.24942855
	MMS19	Decreased	-1.15200309	1.4958827	2.64788579
	POLL	Decreased	-1.15200309	1.39750593	2.54950902
	MPG	Decreased	-1.08926734	1.34632449	2.43559182
	XPA	Decreased	-1.05889369	1.43210356	2.49099725
	PRKDC	Increased	1.11103131	1.93569179	3.04672311
	LIG1	Increased	1.18903382	1.4729375	2.66197133
	PARP2	Increased	1.18903382	1.43146974	2.62050357
	ATP23	Increased	1.26303441	2.22358941	3.48662382
	FEN1	Increased	1.29278175	1.53262871	2.82541046
	SMUG1	Increased	1.36176836	2.35892269	3.72069105
T98G	DDB2	Decreased	-3.18442457	3.34872199	6.53314656
	RAD50	Decreased	-1.51457317	2.10171372	3.61628689
	NEIL2	Decreased	-1.08926734	1.48504073	2.57430806
	PARP3	Decreased	-1.05889369	1.40742357	2.46631725
	NEIL1	Increased	1.02147973	1.70814295	2.72962268
	XRCC6	Increased	1.04963077	1.37048847	2.42011923
	MRE11	Increased	1.41683974	1.86950541	3.28634515
	NEIL3	Increased	1.51601515	1.91642572	3.43244087
	PRKDC	Increased	1.56559718	2.15745316	3.72305034
	RAD54L	Increased	1.74846123	1.38613705	3.13459828
	ATM	Increased	1.88362082	2.81022904	4.69384986
	MGMT	Increased	6.58541327	1.54931989	8.13473316
LN18	DDB2	Decreased	-2	2.78994915	4.78994915
	XPA	Decreased	-1.59946207	1.40191018	3.00137225
	LIG4	Decreased	-1.55639335	1.33331358	2.88970692
	ATXN3	Increased	1.09085343	1.54302699	2.63388042
	PRKDC	Increased	1.53106949	2.52028076	4.05135026
	MRE11	Increased	1.67355642	1.44226428	3.11582071
	ATM	Increased	1.72246602	2.03428103	3.75674705
	NEIL1	Increased	2.50080205	4.25181197	6.75261403
	MGMT	Increased	8.02336626	2.53670439	10.5600706
U373	DDB2	Decreased	-3.64385619	3.23507702	6.87893321
	PARP3	Decreased	-2.47393119	2.305044	4.77897519
	OGG1	Decreased	-1.88896869	1.35086968	3.23983837
	NEIL2	Decreased	-1.83650127	2.30750559	4.14400686
	POLB	Decreased	-1.43440282	1.81070624	3.24510907
	MGMT	Increased	7.10873353	1.34487117	8.4536047
CCF	CCNO	Decreased	-4.05889369	2.08756537	6.14645906

	POLL	Decreased	-2.47393119	4.00436481	6.47829599
	PARP3	Decreased	-2.47393119	2.34611244	4.82004363
	MMS19	Decreased	-2.32192809	2.09210516	4.41403326
	RAD50	Decreased	-1.73696559	2.10946721	3.8464328
	CDK7	Decreased	-1.59946207	1.72234495	3.32180702
	NEIL2	Decreased	-1.51457317	1.58631474	3.10088791
	XPA	Decreased	-1.51457317	1.55458263	3.0691558
	DDB2	Decreased	-1.28630419	1.8515516	3.13785578
	SLK	Decreased	-1.28630419	1.55363373	2.83993791
	POLB	Decreased	-1.18442457	1.68396672	2.86839129
	MLH3	Increased	1.37851162	1.52712658	2.90563821

Table S11. Differentially expressed genes in GBM cell lines relative to the TMZ-sensitive LN229 cell line

Cell line	Name	Change	Fold change (log2)	p-value (-log10)	Manhattan distance
A172	SMUG1	Decreased	-1.35845397	2.35892269	3.71737666
	FEN1	Decreased	-1.28630419	1.53262871	2.8189329
	ATP23	Decreased	-1.25153877	2.22358941	3.47512818
	LIG1	Decreased	-1.18442457	1.4729375	2.65736207
	PRKDC	Decreased	-1.12029423	1.93569179	3.05598603
	POLB	Increased	1.40053793	1.85349987	3.2540378
	RAD50	Increased	1.49057013	2.09441997	3.5849901
	NEIL2	Increased	1.54596837	2.15577142	3.70173979
	PARP3	Increased	2.0071955	2.19477109	4.20196659
	MLH3	Increased	3.592158	2.54928912	6.14144712
T98G	DDB2	Decreased	-4.05889369	2.10165883	6.16055252
	APEX2	Decreased	-1.55639335	1.6342126	3.19060595
	PARP2	Decreased	-1.21759144	1.91760962	3.13520106
	MPG	Increased	1.06350294	1.67742928	2.74093222
	TREX1	Increased	1.09085343	1.97326251	3.06411594
	MRE11	Increased	1.3448285	2.22826557	3.57309407
	ATM	Increased	2.3729521	3.37778598	5.75073807
	MLH3	Increased	4.18903382	2.6058987	6.79493252
	MGMT	Increased	6.01703108	1.54281474	7.55984582
LN18	DDB2	Decreased	-2.94341647	1.96879358	4.91221005
	NTHL1	Increased	1.0071955	1.80701462	2.81421012
	RFC1	Increased	1.24488706	2.28266242	3.52754948
	TREX1	Increased	1.29278175	1.82425632	3.11703806
	MPG	Increased	1.5360529	3.48545225	5.02150515
	MRE11	Increased	1.59931779	1.51768393	3.11700172
	MMS19	Increased	1.62293035	1.50676508	3.12969543
	PARP3	Increased	1.71808758	2.67345933	4.39154692
	POLL	Increased	1.82781902	2.08666307	3.9144821
	ATM	Increased	2.21412481	2.39663908	4.61076388
	NEIL1	Increased	2.22032995	3.64206515	5.86239511
	MLH3	Increased	4.33485427	3.0259491	7.36080337
	MGMT	Increased	7.45508058	2.53372568	9.98880626
U373	DDB2	Decreased	-4.64385619	2.10684879	6.75070498
	EXO1	Decreased	-2.32192809	2.48932097	4.81124906
	RAD51	Decreased	-2.18442457	2.9266483	5.11107287
	RAD18	Decreased	-1.94341647	2.50751899	4.45093546
	OGG1	Decreased	-1.47393119	1.91825675	3.39218794
	FEN1	Decreased	-1.28630419	2.29869111	3.5849953
	RAD54L	Decreased	-1.28630419	1.77124044	3.05754463
	LIG1	Decreased	-1.25153877	2.09012318	3.34166195
	XRCC5	Decreased	-1.18442457	2.54439389	3.72881846
	NEIL3	Decreased	-1.08926734	1.69877462	2.78804195
	XPA	Increased	1.13750352	2.40120949	3.53871302

	MLH3	Increased	4.54720302	3.2533658	7.80056883
	MGMT	Increased	6.54039931	1.3407876	7.88118691
CCF	CCNO	Decreased	-4.05889369	5.30103	9.35992368
	RAD18	Decreased	-2.39592868	1.84912081	4.24504949
	MMS19	Decreased	-2.32192809	1.38018692	3.70211502
	RAD51C	Decreased	-1.55639335	2.2292216	3.78561495
	ERCC6	Decreased	-1.43440282	1.8944217	3.32882452
	DDB2	Decreased	-1.28630419	1.76439581	3.0507
	XPC	Decreased	-1.21759144	2.09130057	3.308892
	MLH3	Increased	1.37851162	2.24139086	3.61990248

Table S12. Top 20 upregulated differentially expressed proteins in T98G compared with A172 cells under control conditions

Name	ID	p-value	Ratio	Function
O75467	O75467	3.8878E-13	9.49	Mitochondrial complex I assembly factor essential for oxidative phosphorylation; required for NADH dehydrogenase complex biogenesis
AKR1D1	P51857	1.4962E-13	7.95	Aldo-keto reductase; steroid 5-beta-reductase involved in bile acid and steroid hormone metabolism
TNFRSF11B	O00300	1.3861E-11	7.95	Secreted decoy receptor for RANKL; modulates osteoclast differentiation and survival; influences apoptosis and angiogenesis pathways
COL6A3	P12111	2.0396E-11	7.94	Extracellular matrix collagen VI alpha-3 chain; contributes to ECM structure, cell adhesion, and tissue integrity
PRDX2	P32119	2.0364E-12	7.71	Thiol-specific peroxidase; reduces peroxides to protect against oxidative stress and regulates redox signaling
FUBP3	Q96I24	3.9654E-11	7.39	DNA/RNA-binding far upstream element-binding protein; regulates transcription and mRNA stability, including MYC-related control
FKBP7	Q9Y680	3.0439E-11	7.24	Endoplasmic reticulum-localized peptidyl-prolyl cis-trans isomerase and chaperone; involved in protein folding and ER stress responses
ACTN2	P35609	1.1285E-12	7.15	Actin-binding cytoskeletal protein; organizes actin filaments and modulates cell motility and signaling
ALDH3A1	P30838	7.4022E-09	7.07	Aldehyde dehydrogenase; detoxifies reactive aldehydes, contributes to oxidative stress resistance and xenobiotic metabolism
BST2	Q10589	1.8716E-09	6.72	Type II transmembrane protein restricting viral particle release; modulates innate immune signaling and NF-κB activation
TCEAL9	Q9UHQ7	1.1601E-10	6.72	Transcription elongation factor A-like protein; implicated in transcriptional regulation and chromatin-associated processes
FHL1	Q13642	6.943E-11	6.71	LIM domain scaffold protein; regulates transcription, cytoskeletal dynamics, and muscle cell differentiation; context-dependent tumor suppressor
HLA-G	P17693	8.4248E-11	6.54	Non-classical MHC class I molecule; mediates immune tolerance and inhibition of NK and T cell responses

CADM1	Q9BY67	3.7174E-10	6.53	Cell adhesion molecule with tumor suppressor functions; maintains cell-cell adhesion and suppresses invasive signaling
ALDH2	P05091	6.7492E-10	6.51	Mitochondrial aldehyde dehydrogenase; metabolizes acetaldehyde and lipid peroxidation products, supports redox homeostasis
RASGRF2	O14827	2.6073E-10	6.43	Ras-specific guanine nucleotide exchange factor; activates Ras and MAPK signaling in response to calcium/cAMP
CYB5A	P00167	1.9023E-09	6.39	Microsomal cytochrome b5; electron donor for desaturases and P450 enzymes in lipid and steroid metabolism
IGFBP5	P24593	1.5015E-10	6.29	Insulin-like growth factor binding protein; modulates IGF bioavailability and signaling, impacts cell survival and migration
KYNU	Q16719	1.0102E-10	6.17	Kynureninase enzyme in tryptophan-kynurenine pathway; produces anthranilate, affects NAD+ biosynthesis and immune-metabolic signaling
PYCARD	Q9ULZ3	4.7885E-09	6.01	Apoptosis-associated speck-like protein containing a CARD; adaptor for inflammasomes (e.g., NLRP3), mediates caspase-1 activation and pyroptosis

Table S13. Top 20 downregulated differentially expressed proteins in T98G compared with A172 cells under control conditions

Name	ID	p-value	Ratio	Function
KATNAL2	Q8IYT4	3.562E-15	-9.42	Microtubule-severing ATPase, regulates cytoskeleton remodeling and neuronal development
P07477	P07477	5.6905E-05	-9.26	Digestive serine protease, breaks down proteins in the small intestine
KCNMA1	Q12791	6.0163E-11	-9.25	Large-conductance, calcium-activated potassium channel; controls neuronal excitability, smooth muscle tone, and neurotransmitter release
ITGB6	P18564	1.2355E-12	-9.04	Integrin beta-6 subunit; mediates cell adhesion to extracellular matrix proteins, important in wound healing and fibrosis
TKTL2	Q9H0I9	1.8153E-10	-8.6	Transketolase-like enzyme; may participate in the non-oxidative branch of the pentose phosphate pathway
GTF3C4	Q9UKN8	1.5273E-13	-8.16	General transcription factor IIIC subunit; required for RNA polymerase III transcription of tRNA and other small RNAs
NES	P48681	5.3076E-11	-7.86	Intermediate filament protein; marker of neural stem/progenitor cells, regulates cytoskeletal organization
CCDC7	Q96M83	6.4032E-12	-7.71	Coiled-coil domain protein; function not fully characterized, possibly involved in cell cycle regulation
ACTA1	P68133	1.3862E-13	-7.65	Major actin isoform in skeletal muscle; essential for muscle contraction and cytoskeletal integrity
KRT8	P05787	1.2924E-08	-7.54	Intermediate filament protein in simple epithelia; provides structural stability and stress resistance
SLC25A4 (ANT1)	P12235	4.9907E-10	-7.33	Mitochondrial ADP/ATP translocator; exchanges ADP and ATP across the inner mitochondrial membrane
ETNPPL	Q8TBG4	8.6277E-05	-7.32	Ethanolamine-phosphate phospho-lyase; involved in phospholipid metabolism and neurotransmitter regulation
AHNAK2	Q8IVF2	1.13E-10	-7.19	Large scaffold protein; implicated in cell architecture, calcium signaling, and cancer progression
ANXA3	P12429	1.1658E-10	-7.17	Calcium-dependent phospholipid-binding protein; regulates membrane trafficking, inflammation, and angiogenesis
DNAJA4	Q8WW22	1.431E-09	-7.16	Heat shock protein co-chaperone (Hsp40 family); assists protein folding and stress respons
RAB17	Q9H0T7	3.5437E-11	-7.09	Small GTPase; regulates polarized

				membrane trafficking in epithelial cells
PDLIM1	O00151	7.9552E-11	-7.03	PDZ and LIM domain protein; links actin cytoskeleton to signaling pathways, regulates transcription factors like NF-κB
CSPG4 (NG2)	Q6UVK1	7.7615E-12	-6.92	Cell surface proteoglycan; involved in cell adhesion, migration, angiogenesis, and tumor progression
OCIAD2	Q56VL3	5.6474E-12	-6.89	Ovarian carcinoma immunoreactive antigen domain-containing protein; associated with mitochondrial function and cancer biology
PODXL	O00592	2.2435E-10	-6.86	Anti-adhesive sialomucin; maintains podocyte structure in kidney, regulates cell migration and tumor metastasis

Table S14. Top 20 upregulated differentially expressed proteins in T98G compared to A172 cells following TMZ treatment

Name	ID	p-value	Ratio	Function
DNAJA4	Q8WW22	1.6933E-10	8.48	Heat shock protein co-chaperone (Hsp40 family); assists protein folding and stress respons
COL6A3	P12111	9.5801E-12	8.42	Extracellular matrix collagen VI alpha-3 chain; contributes to ECM structure, cell adhesion, and tissue integrity
VPS8	Q8N3P4	1.3326E-12	7.49	Subunit of the CORVET complex; involved in endosomal tethering and fusion, essential for early endosome maturation and trafficking
CDH11	P55287	1.117E-10	7.24	Calcium-dependent cell adhesion molecule; mediates homotypic cell–cell adhesion and plays roles in tissue morphogenesis and tumor progression
CADM1	Q9BY67	1.9965E-10	6.86	Cell adhesion molecule with tumor suppressor functions; maintains cell-cell adhesion and suppresses invasive signaling
PTGR1	Q14914	1.5368E-10	6.8	Enzyme that inactivates leukotriene B4 by converting it to 12-oxo-leukotriene B4; involved in inflammatory response regulation
AKR1B10	O60218	5.2723E-07	6.77	Detoxifies reactive aldehydes and regulates lipid synthesis; implicated in cancer metabolism and chemoresistance
PRDX2	P32119	1.2106E-11	6.71	Thiol-specific peroxidase; reduces peroxides to protect against oxidative stress and regulates redox signaling
TNFRSF11B	O00300	1.2236E-10	6.7	Secreted decoy receptor for RANKL; modulates osteoclast differentiation and survival; influences apoptosis and angiogenesis pathways
FUBP3	Q96I24	1.4269E-10	6.68	DNA/RNA-binding far upstream element-binding protein; regulates transcription and mRNA stability, including MYC-related control
BST2	Q10589	2.167E-09	6.64	Type II transmembrane protein restricting viral particle release; modulates innate immune signaling and NF-κB activation
ALDH3A1	P30838	1.6371E-08	6.63	Aldehyde dehydrogenase; detoxifies reactive aldehydes, contributes to oxidative stress resistance and xenobiotic metabolism
KYNU	Q16719	4.3048E-11	6.6	Kynureninase enzyme in tryptophan-kynurenine pathway; produces

				anthranilate, affects NAD+ biosynthesis and immune-metabolic signaling
VPS13D	Q5THJ4	2.8499E-10	6.48	Involved in mitochondrial clearance and autophagy; mutations linked to movement disorders and mitochondrial dysfunction
ALDH2	P05091	7.5887E-10	6.45	Mitochondrial aldehyde dehydrogenase; metabolizes acetaldehyde and lipid peroxidation products, supports redox homeostasis
FKBP7	Q9Y680	1.5552E-10	6.36	Endoplasmic reticulum-localized peptidyl-prolyl cis-trans isomerase and chaperone; involved in protein folding and ER stress responses
NUB1	Q9Y5A7	3.7326E-05	6.27	Interacts with NEDD8 and ubiquitin-like proteins; promotes proteasomal degradation and regulates cell cycle and apoptosis.
CYB5A	P00167	3.1219E-09	6.14	Microsomal cytochrome b5; electron donor for desaturases and P450 enzymes in lipid and steroid metabolism
TOMM20	Q15388	2.3871E-07	5.92	Mitochondrial import receptor; recognizes and translocates precursor proteins into mitochondria.
UQCC3	Q6UW78	1.8285E-10	5.73	Mitochondrial protein required for complex III assembly and oxidative phosphorylation; contributes to respiratory chain stability

Table S15. Top 20 downregulated differentially expressed proteins in T98G compared to A172 cells following TMZ treatment

Name	ID	p-value	Ratio	Function
USP17L3	A6NCW0	7.0463E-11	-10.1	Predicted to enable cysteine-type deubiquitinase activity. Predicted to be involved in regulation of apoptotic process and regulation of protein stability.
KATNAL2	Q8IYT4	3.7269E-15	-9.39	Microtubule-severing ATPase, regulates cytoskeleton remodeling and neuronal development
AHNAK2	Q8IVF2	1.1839E-11	-8.58	Large scaffold protein; implicated in cell architecture, calcium signaling, and cancer progression
SLC25A4	P12235	9.061E-11	-8.39	Mitochondrial ADP/ATP translocator; exchanges ADP and ATP across the inner mitochondrial membrane
TAGLN	Q01995	2.7576E-10	-8.29	Actin-binding protein involved in smooth muscle differentiation and cytoskeletal organization
CEP72	Q9P209	1.0195E-12	-8.12	Involved in microtubule organization and centrosome function during cell division
KRT8	P05787	7.4646E-09	-7.89	Intermediate filament protein in simple epithelia; provides structural stability and stress resistance
NES	P48681	7.7833E-11	-7.62	Intermediate filament protein; marker of neural stem/progenitor cells, regulates cytoskeletal organization
PHAF1	Q9BSU1	1.6574E-11	-7.48	Poorly characterized; may be involved in transcriptional regulation and chromatin remodeling
P07477	P07477	0.00040393	-7.47	Digestive serine protease, breaks down proteins in the small intestine
IER5	Q5VY09	2.9106E-14	-7.22	Stress-inducible transcriptional regulator involved in cell cycle control and DNA damage response
CLIC3	O95833	7.6696E-10	-7.2	Modulates intracellular chloride transport and is involved in vesicle trafficking and cell migration
MME (CD10)	P08473	7.5441E-09	-7.07	Degrades bioactive peptides and regulates cell signaling in immune and cancer contexts
PDLIM1	O00151	7.8921E-11	-7.04	PDZ and LIM domain protein; links actin cytoskeleton to signaling pathways, regulates transcription factors like NF-κB
STARD9	Q9P2P6	2.5237E-10	-7.04	Mitotic kinesin involved in spindle pole assembly and chromosome segregation
CSPG4	Q6UVK1	7.2995E-12	-6.96	Cell surface proteoglycan; involved in cell adhesion, migration, angiogenesis, and tumor progression
TLE2	Q04725	3.0886E-12	-6.91	Transcriptional corepressor in Notch

				signaling and neuronal development
ANXA3	P12429	3.1347E-10	-6.63	Calcium-dependent phospholipid-binding protein; regulates membrane trafficking, inflammation, and angiogenesis
NT5E (CD73)	P21589	4.9985E-09	-6.62	Catalyzes conversion of AMP to adenosine; modulates immune response and tumor microenvironment
PODXL	O00592	5.4948E-10	-6.39	Anti-adhesive sialomucin; maintains podocyte structure in kidney, regulates cell migration and tumor metastasis

Table S16. Top 20 upregulated differentially expressed proteins in A172 cells following TMZ treatment versus control conditions

Name	ID	p-value	Ratio	Function
TUBA4B	Q9H853	4.6339E-14	10.6	Alpha-tubulin family member; structural component of microtubules involved in cytoskeleton organization and intracellular transport.
OR1M1	Q8NGA1	2.1066E-11	8.65	Olfactory receptor; GPCR mediating odorant detection through G protein–coupled signaling
STMN2	Q93045	6.7966E-11	7.21	Neuronal growth-associated protein regulating microtubule dynamics and neurite outgrowth
ACTN2	P35609	2.7424E-12	6.67	Actin-binding cytoskeletal protein; organizes actin filaments and modulates cell motility and signaling
CFAP100	Q494V2	2.5451E-09	6.31	Predicted: Cilia and flagella associated protein; implicated in axonemal structure and ciliary function
C10orf67	Q8IYJ2	5.9839E-09	6.1	Protein of unknown/poorly characterized function; reported roles include stress response and potential tumor suppressive activities
CCT6B	Q92526	8.2521E-09	6.08	Chaperonin-containing TCP1 subunit; assists folding of cytoskeletal proteins like actin and tubulin
KRT24	Q2M2I5	3.8509E-08	5.92	Type I keratin; intermediate filament protein expressed in stratified epithelia
SLC25A25	Q6KCM7	7.5136E-10	5.46	Electroneutral mitochondrial adenyl nucleotide antiporter that mediates transport of adenyl nucleotides across the inner mitochondrial membrane
GPR107	Q5VW38	1.1996E-10	5.1	Orphan GPCR-like protein involved in endomembrane trafficking and retrograde transport; localized to Golgi/endosomes
PTPN21	Q16825	5.1055E-09	5.03	Non-receptor protein tyrosine phosphatase; modulates signaling pathways including focal adhesion and receptor trafficking
AKAP1	Q92667	4.7436E-08	4.85	Mitochondrial A-kinase anchoring protein; scaffolds PKA and other signaling enzymes to mitochondrial outer membrane to regulate metabolism and apoptosis
ANKS1B	Q7Z6G8	2.3597E-09	4.69	Ankyrin repeat and SAM domain protein; adaptor/scaffold implicated in synaptic signaling and receptor trafficking
LRRK1	Q38SD2	1.6149E-07	4.69	Leucine-rich repeat serine/threonine

				kinase; regulates endosomal trafficking and cytoskeletal dynamics
PGK2	P07205	0.00013656	4.67	Testis-specific phosphoglycerate kinase; glycolytic enzyme converting 1,3-BPG to 3-PG with ATP generation
POLE3	Q9NRF9	2.2723E-09	4.65	DNA polymerase epsilon subunit; chromatin-associated factor contributing to DNA replication and genome stability
ACTBL2	Q562R1	9.0107E-09	4.52	Actin beta-like cytoskeletal protein; participates in actin filament organization and cell motility
RNASEH2C	Q8TDP1	1.2202E-05	4.25	Subunit of RNase H2 complex; removes RNA-DNA hybrids (R-loops) and ribonucleotides misincorporated into DNA
ESRP2	Q9H6T0	2.5974E-07	4.24	Epithelial splicing regulatory protein; controls alternative splicing programs defining epithelial cell identity
SERINC1	Q9NRX5	4.7493E-07	4.22	Serine incorporator; transmembrane protein facilitating incorporation of serine into membrane lipids and modulating membrane composition

Table S17. Top 20 downregulated differentially expressed proteins in A172 cells following TMZ treatment versus control conditions

Name	ID	p-value	Ratio	Function
KCNMA1	Q12791	1.1418E-10	-8.8	Large-conductance, calcium-activated potassium channel; controls neuronal excitability, smooth muscle tone, and neurotransmitter release
AVIL	O75366	2.8695E-10	-7.96	Actin-binding protein involved in cytoskeletal organization, neurite outgrowth, and sensory neuron development
CCDC7	Q96M83	6.6669E-12	-7.69	Coiled-coil domain protein; function not fully characterized, possibly involved in cell cycle regulation
DNAJA4	Q8WW22	7.7362E-10	-7.52	Heat shock protein co-chaperone (Hsp40 family); assists protein folding and stress respons
ETNPPL	Q8TBG4	6.9773E-05	-7.49	Ethanolamine-phosphate phospho-lyase; involved in phospholipid metabolism and neurotransmitter regulation
CDH24	Q86UP0	9.346E-10	-7.15	Calcium-dependent cell adhesion molecule; contributes to cell–cell adhesion in epithelial tissues
RAB17	Q9H0T7	4.491E-11	-6.96	Small GTPase; regulates polarized membrane trafficking in epithelial cells
ITGB6	P18564	3.9396E-11	-6.9	Integrin beta-6 subunit; mediates cell adhesion to extracellular matrix proteins, important in wound healing and fibrosis
CABP7	Q86V35	3.3967E-11	-6.34	Regulates calcium-dependent signaling; modulates Golgi-to-plasma membrane trafficking and inhibits PI4KIIIβ activity.
GTF3C4	Q9UKN8	1.1032E-11	-5.85	General transcription factor IIIC subunit; required for RNA polymerase III transcription of tRNA and other small RNAs
TOMM20	Q15388	2.7116E-07	-5.85	Mitochondrial import receptor; recognizes and translocates precursor proteins into mitochondria.
ZYG11B	Q9C0D3	8.8025E-10	-5.55	Cell cycle regulator; part of E3 ubiquitin ligase complex involved in protein degradation and mitotic progression
UQCC3	Q6UW78	4.4247E-10	-5.34	Mitochondrial protein involved in complex III assembly and oxidative phosphorylation; contributes to respiratory chain stability
PRX	Q9BXM0	2.9266E-10	-5.33	Myelination-related protein; maintains

				peripheral nerve structure and Schwann cell cytoskeleton
IQGAP2	Q13576	3.5972E-11	-5.11	Scaffold protein regulating cytoskeletal dynamics, cell adhesion, and signal transduction; interacts with actin and small GTPases
TEF	Q10587	5.1295E-09	-4.91	Transcription factor involved in circadian rhythm regulation and gene expression control
MYH11	P35749	7.2797E-09	-4.74	Smooth muscle myosin; essential for contractile function in vascular and visceral smooth muscle
TMEM17	Q86X19	2.6814E-06	-4.72	Transmembrane protein associated with ciliogenesis and centrosome function
PTBP2	Q9UKA9	3.4233E-08	-4.67	RNA-binding protein regulating alternative splicing in neuronal development
INTS1	Q8N201	3.2919E-08	-4.5	Component of the Integrator complex; involved in snRNA processing and transcriptional regulation

Table S18. Top 20 upregulated differentially expressed proteins in TA98 cells following TMZ treatment versus control conditions

Name	ID	p-value	Ratio	Function
OR1M1	Q8NGA1	2.2608E-12	10.3	Olfactory receptor; GPCR mediating odorant detection through G protein–coupled signaling
TKTL2	Q9H0I9	6.5373E-11	9.32	Transketolase-like enzyme; may participate in the non-oxidative branch of the pentose phosphate pathway
VPS8	Q8N3P4	1.7887E-13	8.75	Subunit of the CORVET complex; involved in endosomal tethering and fusion, essential for early endosome maturation and trafficking
TUBA4B	Q9H853	6.9347E-13	8.63	Alpha-tubulin family member; structural component of microtubules involved in cytoskeleton organization and intracellular transport.
DNAJA4	Q8WW22	2.9336E-10	8.12	Heat shock protein co-chaperone (Hsp40 family); assists protein folding and stress response
NUB1	Q9Y5A7	6.5086E-06	7.44	Interacts with NEDD8 and ubiquitin-like proteins; promotes proteasomal degradation and regulates cell cycle and apoptosis.
VPS13D	Q5THJ4	7.9747E-10	5.97	Involved in mitochondrial clearance and autophagy; mutations linked to movement disorders and mitochondrial dysfunction
INPP5F	Q9Y2H2	2.2838E-10	5.6	Regulates phosphoinositide signaling; involved in cardiac function and axon growth.
MICAL2	O94851	1.377E-11	5.49	Modulates actin cytoskeleton via oxidation; involved in cell morphology and migration
SNAPC4	Q5SXM2	1.2843E-09	5.46	Transcription factor for snRNA genes; part of the SNAPc complex
FUNDC2	Q9BWH2	6.1339E-09	5.39	Mitochondrial membrane protein; may regulate cell death and stress responses
MANSC1	Q9H8J5	1.0696E-08	5.34	Poorly characterized; contains a MANSC domain, possibly involved in cell adhesion or signaling
CGN	Q9P2M7	6.4382E-11	5.25	Tight junction protein; regulates junction assembly and epithelial barrier function
COG8	Q96MW5	6.0382E-09	4.89	Golgi complex subunit; essential for glycoprotein trafficking and Golgi structure
PPP1R21	Q6ZMI0	8.1076E-10	4.81	Regulates PP1 phosphatase activity; involved in endosomal trafficking
TNIP2	Q8NFZ5	9.1188E-09	4.74	Inhibits NF-κB signaling; modulates inflammation and immune responses
MRPS14	O60783	0.00016319	4.7	Component of mitochondrial ribosome;

				involved in mitochondrial protein synthesis
NFKB2	Q00653	1.2783E-10	4.7	Transcription factor regulating immune response, inflammation, and cell survival
ATG4B	Q9Y4P1	3.7555E-09	4.64	Cleaves ATG8 family proteins; essential for autophagosome formation and recycling
DENND2B	P78524	2.2844E-08	4.6	Guanine nucleotide exchange factor (GEF) for Rab GTPases; regulates vesicle trafficking

Table S19. Top 20 downregulated differentially expressed proteins in TA98 cells following TMZ treatment versus control conditions

Name	ID	p-value	Ratio	Function
CEP72	Q9P209	2.1236E-13	-9.17	Involved in microtubule organization and centrosome function during cell division
O75467	O75467	7.5693E-13	-9.02	Mitochondrial complex I assembly factor essential for oxidative phosphorylation; required for NADH dehydrogenase complex biogenesis
IER5	Q5VY09	6.8389E-15	-8.07	Stress-inducible transcriptional regulator involved in cell cycle control and DNA damage response
AKR1D1	P51857	3.7161E-13	-7.41	Aldo-keto reductase; steroid 5-beta-reductase involved in bile acid and steroid hormone metabolism
TMEM123	Q8N131	6.2019E-11	-7.01	Glycosylated transmembrane receptor that induces oncosis via membrane injury; mediates oncotic cell death
RASGRF2	O14827	1.2382E-10	-6.82	Ras-specific guanine nucleotide exchange factor; activates Ras and MAPK signaling in response to calcium/cAMP
HLA-G	P17693	9.2189E-11	-6.5	Non-classical MHC class I molecule; mediates immune tolerance and inhibition of NK and T cell responses
TCEAL9	Q9UHQ7	4.0111E-10	-6.1	Transcription elongation factor A-like protein; implicated in transcriptional regulation and chromatin-associated processes
STARD9	Q9P2P6	4.1026E-09	-5.63	Mitotic kinesin involved in spindle pole assembly and chromosome segregation
USP17L3	A6NCW0	1.7002E-07	-5.34	Predicted to enable cysteine-type deubiquitinase activity. Predicted to be involved in regulation of apoptotic process and regulation of protein stability
OR5K4	A6NMS3	1.3511E-09	-5.33	Olfactory receptor (GPCR) that binds odorant molecules to initiate smell perception
ICA1	Q05084	6.5323E-10	-5.32	Contains arfaptin domain; involved in neurotransmitter secretion and acts as autoantigen in type I diabetes and Sjögren’s syndrome
GSDMA	Q96QA5	2.6736E-08	-5.23	Gasdermin family protein; forms membrane pores upon cleavage, triggering pyroptosis and bacterial defense
AP1S2	P56377	5.3666E-10	-5.02	Small subunit of AP-1 complex; mediates clathrin recruitment and sorting signal recognition in transmembrane receptors
P01880	P01880	6.8112E-10	-4.98	Delta region of immunoglobulin heavy

				chains; involved in antigen binding and regulation of immune responses
PRX	Q9BXM0	7.3645E-10	-4.95	Myelination-related protein; maintains peripheral nerve structure and Schwann cell cytoskeleton
TDRD15	B5MCY1	8.9316E-06	-4.88	Contains Tudor domains; associated with nucleic acid binding and hydrolase activity; linked to PGK1 deficiency
CCDC144 A	A2RUR9	1.3985E-08	-4.84	Coiled-coil domain protein; may inhibit calcium oxalate crystallization and prevent kidney stone formation
CCDC33	Q8N5R6	3.006E-08	-4.64	Coiled-coil protein possibly localized to peroxisomes; associated with microphthalmia and aortic valve prolapse
SYNE1	Q8NF91	0.0001338 5	-4.55	Spectrin-repeat protein at nuclear envelope; links nucleus to cytoskeleton; part of nesprin family

Table S20. p53 status in GBM cell lines

Cell line	TP53 mutation	p53 functional status	References[a]
A172	WT	WT	Van Meir et al., 1994
LN229	p.Lys164Glu (K164E), exon 5 (heterozygous)	WT	Van Meir et al., 1994
T98G	p.Met237Ile (M237I), exon 7	Mutant	Ishii et al., 1999
LN18	p.Cys238Ser (C238S), exon 7	Mutant	Ishii et al., 1999
U373 MG	p.Arg273His (R273H), exon 8	Mutant	Van Meir et al., 1994
CCF-STTG1	WT	WT	Johannessen et al., 2013; Rajan et al., 2016; Dimitriadi et al., 2008.

[a] References:

Van Meir, E. G.; Kikuchi, T.; Tada, M.; Li, H.; Diserens, A. C.; Wojcik, B. E.; Huang, H. J.; Friedmann, T.; de Tribolet, N.; Cavenee, W. K., Analysis of the p53 gene and its expression in human glioblastoma cells. *Cancer Res* 1994, 54, (3), 649-52.

Ishii, N.; Maier, D.; Merlo, A.; Tada, M.; Sawamura, Y.; Diserens, A. C.; Van Meir, E. G., Frequent co-alterations of TP53, p16/CDKN2A, p14ARF, PTEN tumor suppressor genes in human glioma cell lines. *Brain Pathol* 1999, 9, (3), 469-79.

Johannessen, T. C.; Prestegarden, L.; Grudic, A.; Hegi, M. E.; Tysnes, B. B.; Bjerkvig, R., The DNA repair protein ALKBH2 mediates temozolomide resistance in human glioblastoma cells. *Neuro Oncol* 2013, 15, (3), 269-78.

Dimitriadi, M.; Poulogiannis, G.; Liu, L.; Backlund, L. M.; Pearson, D. M.; Ichimura, K.; Collins, V. P., p53-independent mechanisms regulate the P2-MDM2 promoter in adult astrocytic tumours. *Br J Cancer* 2008, 99, (7), 1144-52.

Rajan, T. S.; De Nicola, G. R.; Iori, R.; Rollin, P.; Bramanti, P.; Mazzon, E., Anticancer activity of glucomoringin isothiocyanate in human malignant astrocytoma cells. *Fitoterapia* 2016, 110, 1-7.

Table S21. siRNAs used for cell transfection

Primer	Sequence (5´-3´)
**DDB2-siRNA**	GCCGAUACCCAGAUCCUAA
**con-siRNA**	UGGUUUACAUGUUUUCCUA

	1	2	3	4	5	6	7	8	9	10	11	12
A	APEX1	APEX2	ATM	ATR	ATXN3	BRCA1	BRCA2	BRIP1	CCNH	CCNO	CDK7	DDB1
B	DDB2	DMC1	ERCC1	ERCC2	ERCC3	ERCC4	ERCC5	ERCC6	ERCC8	EXO1	FEN1	LIG1
C	LIG3	LIG4	MGMT	MLH1	MLH3	MMS19	MPG	MRE11A	MSH2	MSH3	MSH4	MSH5
D	MSH6	MUTYH	NEIL1	NEIL2	NEIL3	NTHL1	OGG1	PARP1	PARP2	PARP3	PMS1	PMS2
E	PNKP	POLB	POLD3	POLL	PRKDC	RAD18	RAD21	RAD23A	RAD23B	RAD50	RAD51	RAD51B
F	RAD51C	RAD51D	RAD52	RAD54L	RFC1	RPA1	RPA3	SLK	SMUG1	TDG	TOP3A	TOP3B
G	TREX1	UNG	XAB2	XPA	XPC	XRCC1	XRCC2	XRCC3	XRCC4	XRCC5	XRCC6	XRCC6BP 1
H	ACTB	B2M	GAPDH	HPRT1	RPLP0	HGDC	RTC	RTC	RTC	PPC	PPC	PPC

Figure S1. Schematic of DNA repair RT^2 Profiler PCR array and internal controls. The array includes 84 genes associated with DNA repair pathways (rows A–G). Five genes were evaluated as potential housekeeping controls (H1–H5). Internal controls comprised human genomic DNA contamination (HGDC; H6), reverse transcription control (RTC; H7–H9), and positive PCR controls (PPC; H10–H12).

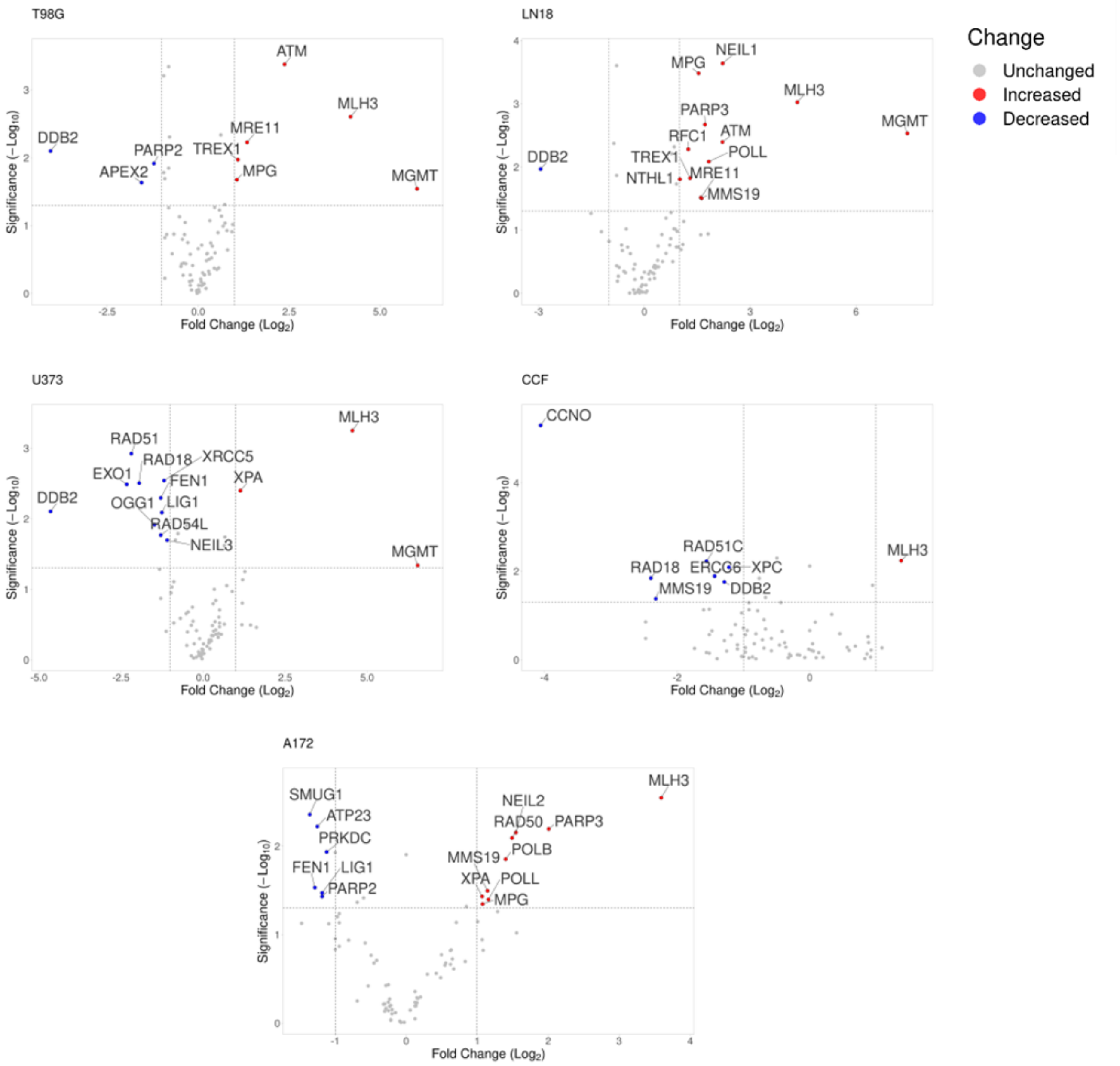

Figure S2. Differential gene expression in GBM cell lines relative to the TMZ-sensitive LN229 cell line. Volcano plots of differentially expressed genes (DEGs) in the different pairwise comparisons. Genes with fold change < 2 and $p < 0.05$ are shown in blue (downregulated relative to LN229), whereas genes with fold change > 2 and $p < 0.05$ are shown in red (upregulated relative to LN229). Genes with non-significant changes are shown in gray.

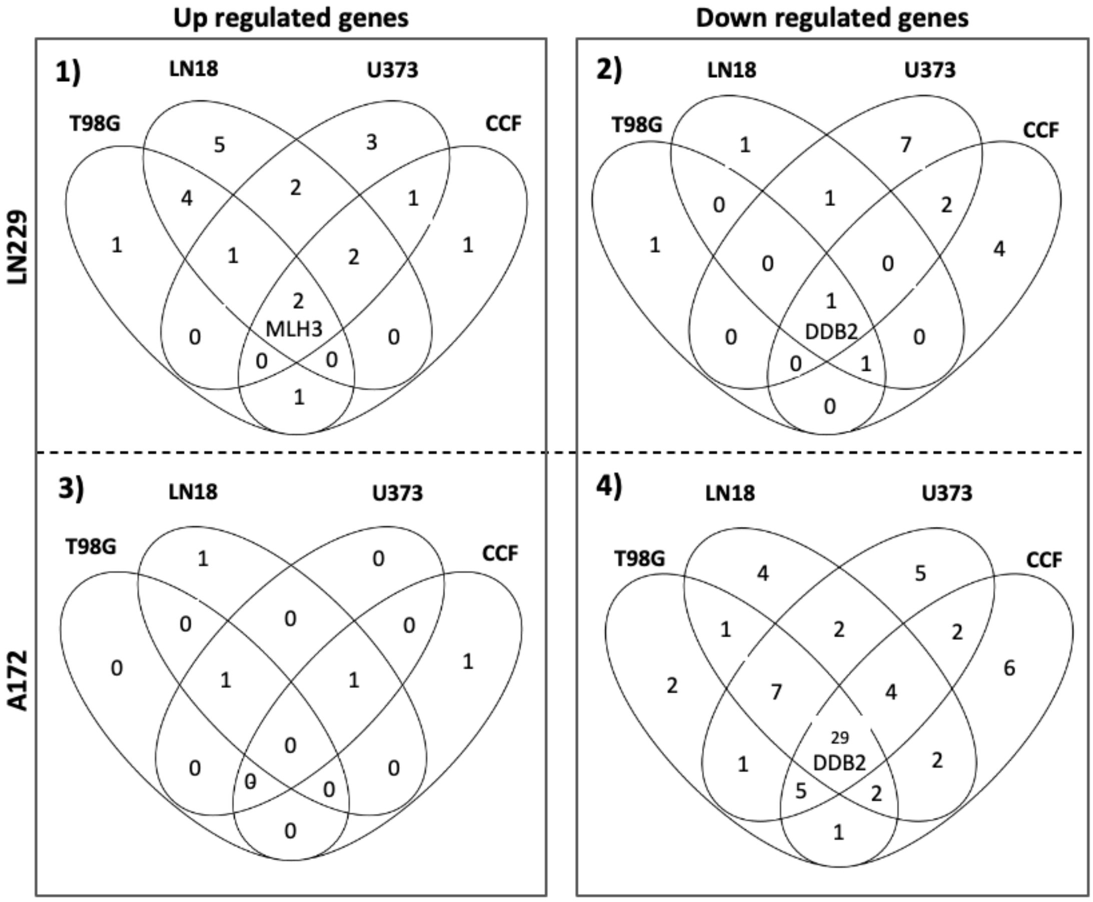

Figure S3. Differential gene expression analysis in GBM cell lines using GAPDH as the normalization control. Venn's diagrams showing the overlap of differentially expressed genes (DEGs) between TMZ-resistant cell lines (T98G, LN18, U373 and CCF) and TMZ-sensitive cell lines LN229 (top) and A172 (bottom). DEGs were identified using the same fold-change and statistical significance criteria applied in the analysis shown in Figure 2B.

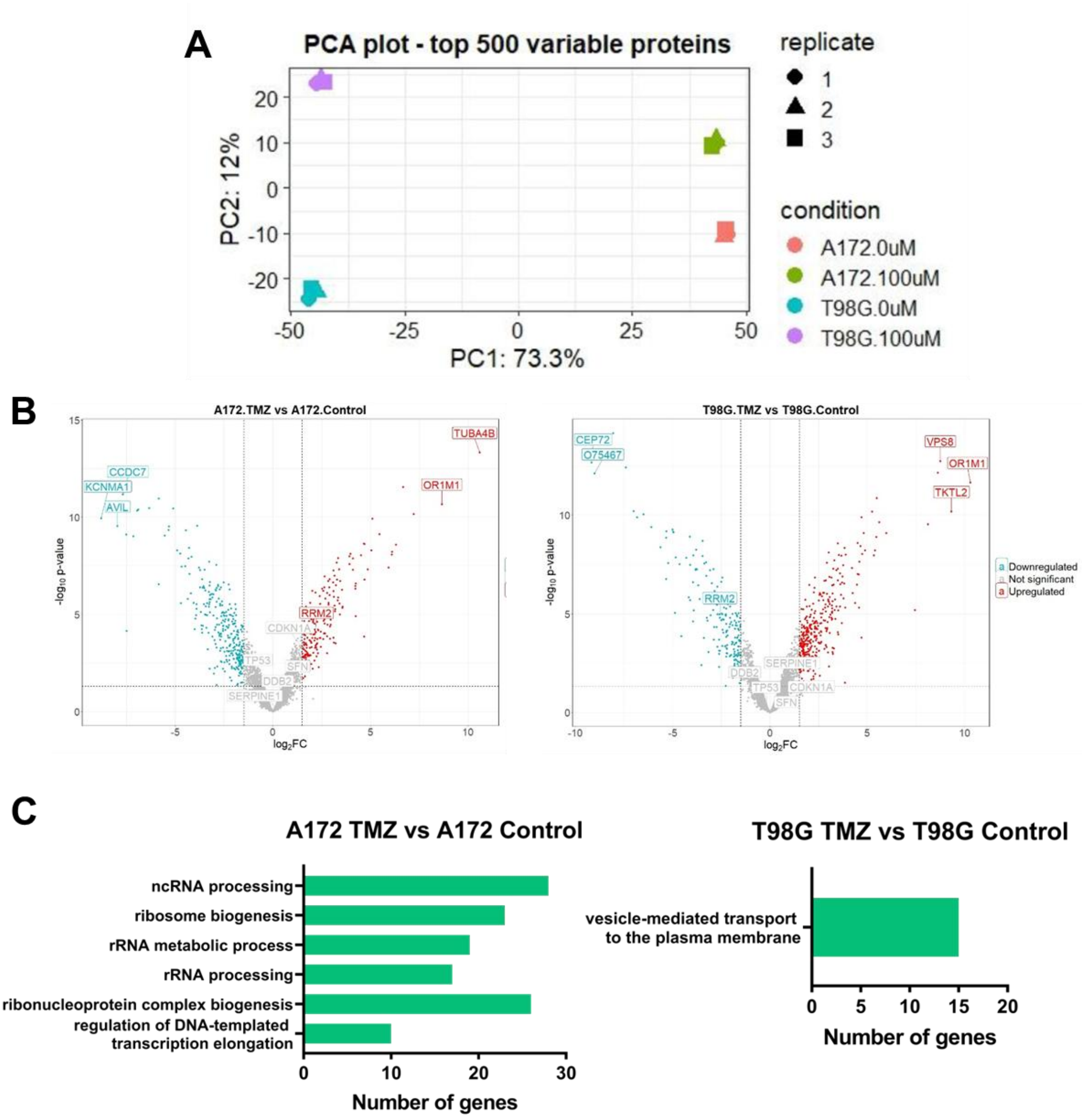

Figure S4. Proteomic analysis of TMZ response in GBM cell lines. (A) Principal component analysis (PCA) of the 500 most variable proteins across three biological replicates per group. (B) Volcano plots of differentially expressed proteins (DEPs) in A172 (left) and T98G (right) cells comparing TMZ-treated versus control conditions. Significantly upregulated proteins ($\log_2$ fold change > 1.5, $p < 0.05$) are shown in red, significantly downregulated proteins ($\log_2$ fold change < −1.5, $p < 0.05$) in blue, and proteins with non-significant changes in gray. (C) GO enrichment analysis of DEPs in A172 and T98G cells following TMZ treatment. Significant GO terms ($p < 0.05$) are shown on the y-axis with corresponding gene counts on the x-axis.
